# Amphiphilic Cationic Carbon Dots for Efficient Delivery of Light‐Dependent Herbicide

**DOI:** 10.1002/advs.202406523

**Published:** 2024-08-21

**Authors:** Gang Tang, Jialu Wang, Jianhua Xiao, Yulu Liu, Yuqi Huang, Zhiyuan Zhou, Xiaohong Zhang, Gaohua Hu, Weiyao Yan, Yongsong Cao

**Affiliations:** ^1^ College of Plant Protection China Agricultural University NO. 2 Yuanmingyuan West Road Beijing 100193 China

**Keywords:** activity, carbon dots, herbicide, light conversion, protoporphyrinogen oxidase

## Abstract

The inefficient delivery of herbicides causes unpleasant side effects on the ecological environment. Protoporphyrinogen oxidase (PPO)‐inhibiting herbicides rely on the presence of external light to exert the activities and thus their performance in the field is extremely susceptible to the light environment. Here, taking acifluorfen (ACI) as a model PPO‐inhibiting herbicide to enhance efficacy by boosting the utilization rate of sunlight, amphiphilic cationic CDs (CPC‐CDs) from cetylpyridinium chloride (CPC) as a precursor, is first prepared as a supplementary light source generator, and subsequently co‐assembled with ACI through non‐covalent bond interactions to obtain the stable fluorescent nanoparticles (ACI@CPC‐CDs). ACI@CPC‐CDs with fascinating physicochemical properties can penetrate the leaves of weeds through the stomata and undergo a long‐distance transport in the cell intervals. Under low light intensity, CPC‐CDs can be applied as the internal light source to promote the formation of more singlet oxygen to damage the leaf cell membrane, consequently improving the herbicidal activity of ACI. Moreover, the safety evaluation of ACI@CPC‐CDs demonstrates no risk to non‐target organisms and the environment. Therefore, this work offers a promising strategy for the efficient delivery of light‐dependent PPO‐inhibiting herbicides and opens new insights into the application of CDs in the development of sustainable agriculture.

## Graphical abstract

Amphiphilic cationic CDs derived from the surfactant cetylpyridinium chloride are prepared via a simple, green, and time‐saving way, which are used as a supplementary light source generator to fabricate a co‐assembled nano‐system with the typical protoporphyrinogen oxidase‐inhibitor acifluorfen through noncovalent molecular recognition for improving herbicidal activities of this kind of herbicides and mitigating the environmental risks.

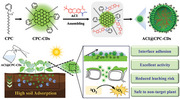

## 1 Introduction

The presence of weeds in the field seriously restrains the yield and quality of crops by competing for nutrients, water, light, and living space, posing a grave threat to food security under the severe situation of global population expansion.^[^ ^1^ ^]^ Chemical herbicides with low cost, rapid action, desirable bioactivity, and convenient operation provide solutions for weed management and more than a million tons of herbicides are pumped into most crop production systems in modern agriculture to control weeds each year.^[^ ^2^ ^]^ Unfortunately, large amounts of herbicides are lost from the site of the target object in the application due to the low utilization rates caused by the shortcomings (e.g., weak interface adhesion,^[^ ^3^ ^]^ volatilization,^[^ ^4^ ^]^ leaching,^[^ ^5^ ^]^ and instability^[^ ^6^ ^]^) of the physicochemical properties of the active ingredients (AIs), which not only increases the cost of crop production from excessive use of herbicides but also brings tremendous risks to human health and ecosystems after the herbicides leak into the environment.^[^ ^7^ ^]^ Given these problematic situations of herbicide application, searching for an efficient and environmentally friendly strategy to deliver herbicides is highly essential and urgent but faces a huge challenge.

Protoporphyrinogen oxidase (PPO, EC 1.3.3.4)‐inhibitors featuring the advantages of low dosage, excellent efficacy, good crop selectivity, broad herbicidal spectrum, and controllable resistance risk, an important branch of herbicides, have been widely used in crop fields to decimate weeds around the world since the first PPO‐inhibiting herbicide was commercialized in 1963.^[^ ^8^ ^]^ After more than half a century of development, PPO‐inhibiting herbicides involving ≈30 compounds in 9 classifications including diphenyl ethers, phenyl pyrazoles, thiadiazoles, and so on have been discovered.^[^ ^9^ ^]^ As the last key enzyme in the chlorophyll and heme biosynthetic pathway, PPO could catalyze the oxygen‐dependent oxidation of protoporphyrinogen IX (Protogen IX) to form protoporphyrin IX (Proto IX).^[^ ^10^ ^]^ When the susceptible plants were treated with PPO‐inhibiting herbicides, large quantities of Protogen IX get accumulated in the chloroplast and then permeates into the cytoplasm to be converted to Proto IX by an auto‐oxidation process.^[^ ^11^ ^]^ In the presence of sunlight, Proto IX leads to the occurrence of numerous reactive oxygen species, resulting in the degradation of cell membranes and cell lysis and subsequent death of the treated plants.^[^ ^12^ ^]^ Based on the mechanism of action of PPO‐inhibiting herbicides, it could be found that the performance of herbicidal activities has a close relationship with the accessibility, intensity, and wavelength of light.^[^ ^13^ ^]^ Studies have observed that the wavelengths of light between 415 and 615 nm were more necessary than that less than 410 nm for the activities of PPO inhibitors.^[^ ^14^ ^]^ The previous works revealed that the irradiation with low light intensity (PAR_max_ = 140 µmol m^−2^ s^−1^) could cause poor activity for PPO inhibitors and the irradiation environment of the weeds was subject to the crop canopy and the weather, thus having a significant effect on the efficacy of PPO inhibitors under field conditions.^[^ ^15^ ^]^ Therefore, considering the particularity for light utilization of PPO‐inhibiting herbicides, it is urgent to design a new formulation for these herbicides to enhance efficacies by improving the utilization rate of sunlight.

Carbon dots (CDs) defined as carbon‐based and quasi‐spherical nanomaterials with a size below 10 nm in diameter have attracted immense attention in many fields on account of their highly tunable photoluminescence, excellent water dispersibility, negligible toxicity, high biocompatibility, and low cost.^[^ ^16^ ^]^ CDs with outstanding optical properties have been explored for application to adjust plant photosynthesis in agriculture and some remarkable progress has been made in this area, which proved that small‐size CDs can pass the barrier of the leaf bio‐interface to enter the plant system through the stomatal pathway after foliar spray and are endocytosed by intact plant cells and accumulated in the cytoplasm, a portion of which gets further into the chloroplasts and cell nucleus to form CDs based complex system.^[^ ^17^ ^]^ With the aid of strong absorption centered in the ultraviolet (UV) region of CDs and their electron transfer capability as a good electron donor as well as acceptor, the formed complex system between CDs and chloroplasts can accelerate light conversion and energy transfer to promote photosynthetic activity and efficiency.^[^ ^18^ ^]^ CDs prepared by using citric acid and ethanolamine as the precursors can convert UV radiation (300–370 nm) to photosynthetic active radiation (370–500 nm) after compounding with the chloroplasts and thus significantly enhance photosynthesis.^[^ ^19^ ^]^ Aggregation‐induced emission CDs obtained by the hydrothermal treatment of natural quercetin can be used as an optical amplifier to efficiently harvest ultraviolet light to improve the electron transport rate in the complex photosystem II and display a higher photosynthetic activity than that of natural chloroplasts.^[^ ^20^ ^]^ Hence, in light of the peculiarity of CDs in photosynthesis, it is unambiguous that CDs can be absorbed, transported, and accumulated by plants, and their fluorescence characteristics can also play a light converter to make plants full use of solar radiation.

Acifluorfen (ACI) (5‐[2‐chloro‐4‐(trifluoromethyl)‐phenoxy]−2‐nitrobenzoic acid), a typical PPO inhibitor, has been widely applied for selectively controlling broad‐leaved weeds in soybean and peanut cropping systems.^[^ ^21^ ^]^ Undoubtedly, this herbicide requires light for phytotoxicity, and its activities were easily interfered by light intensity.^[^ ^22^ ^]^ In addition, as a weak acid (p*K*a = 3.50), ACI was usually processed into the form of sodium salt in commercial formulations.^[^ ^23^ ^]^ However, ACI sodium salt with a high‐water solubility (>200 000 mg L^−1^) has strong mobility in soil (leaching potential index = 3.15), which increases the risks of contamination to ground and surface water.^[^ ^24^ ^]^ In this work, to improve herbicidal activities and mitigate environmental risks of PPO‐inhibiting herbicides by the optimization of physicochemical properties and the increase of sunlight utilization, amphiphilic cationic CDs (CPC‐CDs) from the surfactant cetylpyridinium chloride (CPC) as a precursor, were First prepared via a simple, green, and time‐saving way at room temperature, and subsequently co‐assembled with ACI through non‐covalent bond interactions to obtain the stable nanoparticles (ACI@CPC‐CDs).

## 2 Results and Discussion

### 2.1 Synthesis and Characterization of CPC‐CDs

Due to the high carbon content and nitrogen‐containing heteroaromatic functionality, the surfactant CPC has been used as a promising precursor for the synthesis of cationic CDs. Since the first preparation of fluorescent CDs with tunable photoluminescence using hydrothermal treatment of CPC at 150 °C, a series of water‐soluble or organic soluble CDs based on CPC have been synthesized by altering the concentration of sodium hydroxide (NaOH) and reaction time at room temperature without additional energy input.^[^ ^25^ ^]^ However, the methods for synthesis of the CPC‐based CDs were either high energy consumption or long reaction time, which was not suitable for the large‐scale synthesis to be applied in agriculture. In this study, CPC‐based CDs with different physicochemical properties were prepared by controlling the ultrasonic exposure time of the reaction systems. Figure S1a (Supporting Information) shows CPC aqueous solutions containing NaOH after different ultrasonic exposure time. It can be found that with the treated time prolongation, the solutions turned from colorless to deep yellow, indicating that more ultrasonic exposure time could facilitate the carbonization of CPC more effectively. Moreover, the corresponding solutions displayed fluorescence under ultraviolet light centered at 365 nm and the fluorescence intensity gradually increased with the ultrasonic time, attributing to the formation of CDs in the solution (Figure S1b, Supporting Information).

**Figure 1:**
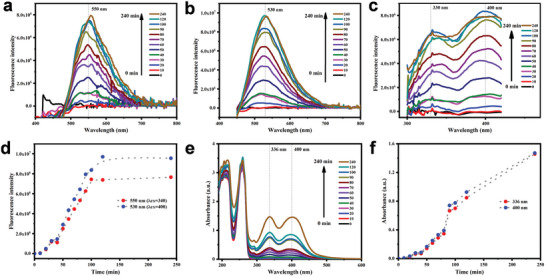
Optical characterizations of CPC aqueous solutions containing NaOH after different ultrasonic exposure times. All the tested solutions were obtained by diluting the initial CPC reaction solutions 10 times with ultrapure water. Emission spectra of the CPC reaction solutions at an excitation wavelength of 340 nm (a) and 400 nm (b). Excitation spectra of the CPC reaction solutions at an emission wavelength of 520 nm (c). The fluorescence intensities at 550 nm (λ_ex_ = 340 nm) and 530 nm (λ_ex_ = 400 nm) of the CPC reaction solutions (d). UV‐vis spectra of the CPC reaction solutions (e). The UV‐vis absorption intensities of the CPC reaction solutions at 336 and 400 nm (f).

**Figure** **1** shows the optical characterizations of CPC aqueous solutions containing NaOH after different ultrasonic exposure times. At an excitation wavelength of 340 nm, the maximum emission wavelength of the solutions was ≈550 nm and the intensity increased as the time increased (Figure 1a). When the exciting wavelength was 400 nm, there was a fluorescence peak for the reaction solution at ≈530 nm and the increase of fluorescent intensity over time was also observed (Figure 1b). Figure 1c displays excitation spectra of the CPC reaction solutions at an emission wavelength of 520 nm, which indicates that the excitation wavelengths of the solution with CPC‐CDs were ≈330 and 400 nm, respectively. In Figure 1d, an increasing trend of the fluorescence intensities at 550 nm (λ_ex_ = 340 nm) and 530 nm (λ_ex_ = 400 nm) of the CPC reaction solutions exhibited intuitively with time. However, the increase in the fluorescence intensities of the CPC reaction solutions was insignificant after ultrasonic treatment for 120 min. Figure 1e shows the UV‐vis spectra of the CPC reaction solutions. At 0 min, the absorption peaks in the range of 190–300 nm were corresponded to CPC. Under the ultrasonic treatment, the new absorption peaks at 336 and 400 nm occurred and their intensities increased with reaction time, which could be attributed to the formation of CDs. The absorbances at 336 and 400 nm at various reaction times are displayed in Figure 1f. Unlike the fluorescence intensities of the reaction solutions, the absorbances increased significantly with time, suggesting that the new substances were constantly generated.

**Figure 2:**
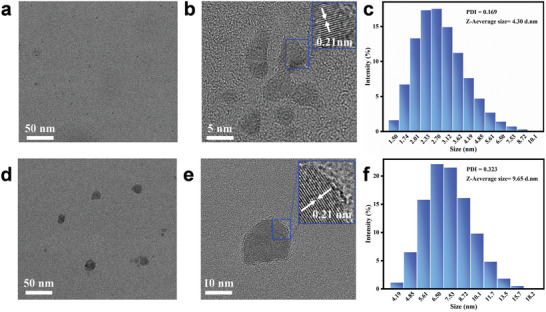
TEM images and particle size distribution of CPC‐CDs obtained after the ultrasonic treatment for 100 min (a,b; c) and 240 min (d,e; f).

**Figure** **2** displays transmission electron microscopy (TEM) images and particle size distribution of CPC‐CDs obtained after the ultrasonic treatment for 100 min and 240 min. According to Figure 2a–c, CPC‐CDs prepared in a short reaction time (100 min) were nearly spherical particles with a lattice spacing of 0.21 nm, a mean size of 4.30 nm, and a low polydispersity index (PDI) value of 0.169, suggesting the obtained CPC‐CDs had a good dispersity with no trace of agglomeration. In Figure 2d–f, CPC‐CDs obtained after the ultrasonic treatment of 240 min still had a lattice spacing of 0.21 nm, but their mean size reached 9.65 nm and had a high PDI value (0.323). These results implied that the growth of the carbon core of CPC‐CDs was continuous with the increase of treated time and the larger size CPC‐CDs presented poor stability. By analyzing the optical properties and particle size distribution of CPC‐CDs of the different reaction solutions, CPC‐CDs obtained at the ultrasonic treatment for 100 min were further characterized and used for the co‐assembled formation with ACI, which was confirmed and complemented in the next experiments.

Figure S2 (Supporting Information) shows that the fluorescence quantum yield of the CPC‐CDs in water was calculated to be 7.24% by using rhodamine B in ethanol as a reference. The Raman spectrum of CPC‐CDs (Figure S3, Supporting Information) showed two peaks at 1324 cm^−1^ (D‐band) corresponding to the sp3 defects in the carbon atoms and 1594 cm^−1^ (G‐band) corresponding to the sp2 hybridized carbon atoms, where the ID/IG ratio could serve as an indicator to analyze the defect concentration in the curved graphite layers of carbon materials.^[^ ^26^ ^]^ The ID/IG of the prepared CPC‐CDs was 1.89, which indicated that the disordered carbon was more than the ordered graphite in the structures of CPC‐CDs. This result may be explained by the presence of a long alkyl chain in the CPC‐CDs. The XRD spectrum of CPC‐CDs can be observed in Figure S4 (Supporting Information). The characteristic peaks were found at 2θ angles of 22.8 and 42.3°, which corresponded to the (002) and (100) crystallographic planes, respectively.^[^ ^26a^ ^]^ The average interlayer spacing (0.39 nm) of the (002) plane (d_002_) was slightly higher than that of pure graphite, mainly ascribing to the effect of the functional group on the surface of CPC‐CDs.^[^ ^27^ ^]^ The average interlayer spacing (0.21 nm) of the (100) plane (d_100_) was consistent with the results of TEM. Figure S5 (Supporting Information) shows the critical micelle concentration value of CPC‐CDs in an aqueous solution is only 41.1 mg L^−1^, which indicates that CPC‐CDs could form micelles in water due to amphipathy. Based on these results, it can be concluded that small‐sized amphiphilic CPC‐CDs with unique optical properties were successfully prepared.

### 2.2 Preparation of ACI@CPC‐CDs

Figure S6 (Supporting Information) presents self‐assembly phenomena between CPC aqueous solutions containing NaOH after different ultrasonic exposure times and ACI sodium salt solution. According to the results, the carboxylate group of ACI sodium salt could interact with the pyridine group of the CPC that was not transformed CDs in the reaction solutions (ultrasonication for 0–30 min) to produce the water‐insoluble ionic complex. With the increasing of the treated time, the self‐assembly phenomena presented by the yellow micellar solution became more apparent (ultrasonication for 40–100 min). However, the deep‐yellow oily liquid occurred after the ultrasonic treatment for 120 min, which suggested that the hydrophobicity of the generated CPC‐CDs was enhanced gradually and thus the co‐assembly between CPC and ACI was broken. Therefore, the CPC‐CDs obtained at the ultrasonic treatment for 100 min were the optimal choice to form co‐assembly with ACI. Moreover, the effect of the added amount of ACI sodium salt on the formation of co‐assembly with CPC‐CDs is displayed in Figure S7 (Supporting Information). Along with the increase in the concentration of ACI sodium salt, the color of the different reaction systems gradually deepened. When the concentration of ACI sodium salt was 24 000 mg L^−1^, the appearance of stratification phenomena meant that the colloidal solution was unstable. The change of fluorescence intensity of the corresponding emission spectra of different reaction systems also proved the interaction between ACI sodium salt and CPC‐CDs. Considering the maximum mass of ACI in the ACI@CPC‐CDs nanocomplex, the optimal mass ratio of ACI to CPC‐CDs was selected as 1:2. The *AR* (assembled rate) of ACI@CPC‐CDs determined by high‐performance liquid chromatography was 99.17%, indicating that almost all ACI could be bounded to CPC‐CDs under the ratio. According to the optimal mass ratio of ACI to CPC‐CDs and the *AR* of ACI@CPC‐CDs, the loading content of ACI in the co‐assembly was calculated to be 33.15%.

### 2.3 Morphological Characterization of ACI@CPC‐CDs

**Figure 3:**
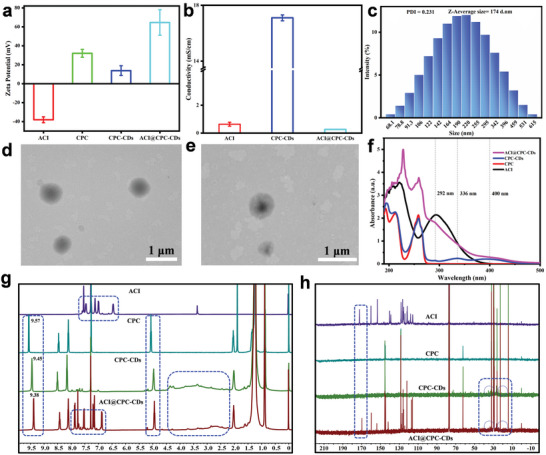
TEM images (a,b) and particle size distribution (c) of ACI@CPC‐CDs. Zeta potentials (d), conductivities (e), UV‐vis absorbance spectra (f), ^1^H NMR spectra (g), and ^13^C NMR spectra (h) of ACI, CPC, CPC‐CDs, and ACI@CPC‐CDs.

As mentioned in the introduction, ACI sodium salt with high water solubility is easily dissolved in water to form a stable and clear solution (Figure S8a, Supporting Information). And ACI sodium salt aqueous solution had strong negative charges (−38.1 mV) and exhibited good electrical conductivity (0.641 mS cm^−1^) (**Figure** **3**a,b). CPC, a cationic surfactant, also had high water solubility (Figure S8b, Supporting Information) and presented strong positive charges (+32 mV). The suspension of CPC‐CDs derived from CPC displayed deep yellow (Figure S8c, Supporting Information) and very high electrical conductivity (17.1 mS cm^−1^). When the co‐assembly based on ACI sodium salt and CPC‐CDs was obtained, the formed ACI@CPC‐CDs suspension (Figure S8d, Supporting Information) with sizes varying from 68.1 to 615 nm (Figure 3c) exhibited an evident Tyndall phenomenon (Figure S8e, Supporting Information), very low conductivity (0.265 mS cm^−1^), and stronger positive charges (+64.6 mV) than CPC. The TEM images of ACI@CPC‐CDs (Figure 3d,e) show nearly spherical particles with an average diameter of ≈500 nm and a core‐shell structure. In the formation process of ACI@CPC‐CDs, positively charged CPC‐CDs could interact with negatively charged ACI sodium salt through electrostatic interaction. Then, the hydrophobic diphenyl ether structure of ACI could aggregate to form a core in the presence of π‐π stacking and other forces. Amphiphilic CPC‐CDs with the hydrophilic groups on the surface were more likely to aggregate as the outer shell, resulting in core shells arranged in an orderly manner dispersing in an aqueous solution.

### 2.4 Spectral Characteristics of ACI@CPC‐CDs

UV‐vis spectroscopy, ^1^H and ^13^C nuclear magnetic resonance (NMR) spectra, Fourier transformation infrared (FTIR) spectroscopy, and high‐resolution X‐ray photoelectron spectroscopy (XPS) spectra were applied to analyze the spectroscopic properties of ACI@CPC‐CDs. The UV‐vis absorption spectra of ACI sodium salt, CPC, CPC‐CDs, and ACI@CPC‐CDs were obtained at 190–500 nm (Figure 3f). According to the results, the characteristic absorption peaks of ACI sodium salt and CPC were located at 292 and 258 nm, respectively. Compared with CPC, the prepared CPC‐CDs had other absorption peaks at 336 and 400 nm. The characteristic absorption peaks in ACI@CPC‐CDs basically kept the features of both ACI sodium salt and CPC‐CDs. However, the characteristic absorption peak at 292 nm for ACI@CPC‐CDs exhibited a slight blue shift and lower intensity than ACI sodium salt.

Figure 3g shows the ^1^H NMR spectra of ACI sodium salt, CPC, CPC‐CDs, and ACI@CPC‐CDs. The results demonstrated that the chemical shifts of protons in ACI sodium salt were at low field region (6–8 ppm). When CPC was transformed into CPC‐CDs, the chemical shifts of protons (‐CH_2_‐ and ‐CH‐) located around the nitrogen atom of the pyridine ring of CPC shifted to higher field strengths. Compared with CPC, some new proton signals at 2.5–4.5 and 6.5–8.0 ppm were found in the CPC‐CDs. These changes in CPC could support the successful synthesis of CPC‐CDs. And there were some pyridine groups on the surface of CPC‐CDs. For the ACI@CPC‐CDs nanocomplex, the distribution of the chemical shifts of protons was basically like those of ACI sodium salt and CPC‐CDs. However, the chemical shifts of protons of CPC‐CDs shifted further to a high field due to the electronic shield effect of the carboxyl group of ACI. According to the ^13^C NMR spectra of the different samples (Figure 3h), it could be concluded that the chemical shifts of carbon of ACI in the ACI@CPC‐CDs shifted to a low field compared with free ACI sodium salt and some new carbon signals at 10–50 ppm were detected.

Figure S9 (Supporting Information) depicts the IR spectra of ACI sodium salt, CPC, CPC‐CDs, and ACI@CPC‐CDs. The results displayed that there were characteristic absorption IR peaks at 1264.28 (aromatic ether, ‐ArOAr), 1326.72 (trifluoromethyl, ‐CF_3_), and 1526.22 (nitro, ‐NO_2_) cm^−1^ for ACI sodium salt. CPC showed the absorption peaks of nitrogen‐containing bonds (1639.53 cm^−1^) and carbon‐hydrogen bonds (2850.09 and 2913.93 cm^−1^). Similar absorption peaks were also observed in CPC‐CDs, uncovering the presence of the pyridinium ring and the aliphatic carbon on the surface of CPC‐CDs. Additionally, the broad absorption peak of CPC‐CDs at ≈3421.45 cm^−1^ could be assigned to the O‐H/N‐H stretching, indicating that CPC‐CDs had a certain hydrophilic property.^[^ ^25b^ ^]^ When ACI sodium salt interacted with CPC‐CDs, the characteristic absorption peaks of the obtained ACI@CPC‐CDs were very similar to those of the two components. These results demonstrated that the formation of ACI@CPC‐CDs was driven by the non‐covalent interactions.

The thermogravimetric (TG) and derivative of thermogravimetry (DTG) curves of ACI sodium salt, CPC, CPC‐CDs, and ACI@CPC‐CDs are shown in Figure S10 (Supporting Information). The TG and DTG curves of ACI sodium salt displayed its mass loss occurred mainly in the temperature range of 200–400 °C. The mass of CPC with poor thermal stability was reduced to almost zero at ≈260 °C. Compared with CPC, CPC‐CDs presented better thermal stability. When the temperature rose to 600 °C, the maas of CPC‐CDs remained at ≈20%. According to the TG and DTG curves of ACI@CPC‐CDs, the mass loss (≈80%) occurred between 200–500 °C. It could be inferred that ACI@CPC‐CDs exhibited good thermal stability and were composed of ACI and CPC‐CDs.

**Figure 4:**
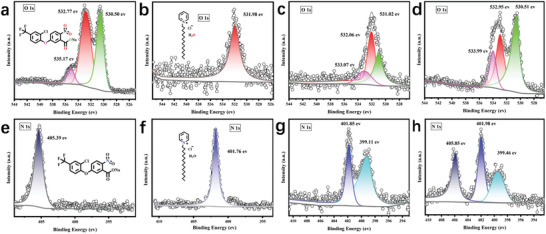
High‐resolution XPS O 1s and N 1s spectra of ACI (a,e), CPC (b,f), CPC‐CDs (c,g), and ACI@CPC‐CDs (d,h).

The high‐resolution XPS was performed to further characterize the chemical components of ACI@CPC‐CDs (**Figure** **4**). The deconvolution of O_1s_ XPS spectra of ACI sodium salt showed three kinds of oxygen bonds, corresponding to ‐C─O at 530.50 eV, ‐N═O at 532.77 eV, and ‐C─O─C‐ at 535.17 eV (Figure 4a). For CPC, the presence of O_1s_ spectrum might be due to the water molecule in the CPC (Figure 4b). After the formation of CPC‐CDs, the O_1s_ spectrum of CPC was deconvoluted into three peaks (533.07, 532.06, and 531.02 eV), implying that the different chemical state oxygens co‐existed on the surface of CPC‐CDs (Figure 4c). For ACI@CPC‐CDs, the O_1s_ region possessed three peaks at 530.51, 532.95, and 533.99 eV (Figure 4d). Due to the presence of the nitro group, the deconvolution of N_1s_ XPS spectrum of ACI sodium salt showed a peak at 405.39 eV (Figure 4e). The N_1s_ spectroscopy of CPC containing pyridinic nitrogen was deconvoluted to the peak at 401.76 eV (Figure 4f). For CPC‐CDs, the N_1s_ spectra were deconvoluted into two peaks (399.11 and 401.85 eV), indicating the generation of new chemical state nitrogen in the CPC‐CDs (Figure 4g). The spectrum of N_1s_ of ACI@CPC‐CDs revealed three different types of nitrogen states (399.46, 401.98, and 405.85 eV) in the nanocomplex. According to Figure 4f–h, the binding energy of N_1s_ was from 401.76 eV (CPC) to 401.85 eV (CPC‐CDs) to 401.98 eV (ACI@CPC‐CDs), which suggested that the chemical state of nitrogen was constantly in the process of change due to the formation of CPC‐CDs and ACI@CPC‐CDs.

**Figure 5:**
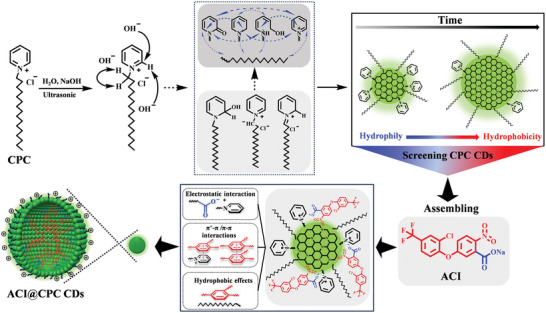
The schematic depiction of CPC‐CDs formation and possible formation mechanism of ACI@CPC‐CDs.

Combined with the zeta potential, conductivity, morphological characterization, spectral characteristics (^1^H and ^13^H NMR spectra, FITR spectra, UV‐vis spectra, and XPS spectra), and thermal behavior of CPC‐CDs and ACI@CPC‐CDs, the possible formation mechanisms of CPC‐CDs and ACI@CPC‐CDs are shown in **Figure** **5**. First, using NaOH as the initiator and ultrasonic as controllable energy input, CPC molecules as the carbon sources were carbonized through multiple reactions such as oxidation, disproportionation, and ring‐opening reactions to form the hydrophilic‐hydrophobic adjustable CPC‐CDs. Then, the PPO inhibitor ACI sodium salt was added and co‐assembled with the CPC‐CDs with different properties through noncovalent molecular recognition. Finally, the stable nanoparticles (ACI@CPC‐CDs) were obtained by strong interacting forces including electrostatic interactions, hydrophobic effect, π‐π stacking, hydrogen bond, and so on.

### 2.5 Physicochemical Property of ACI@CPC‐CDs

**Figure 6:**
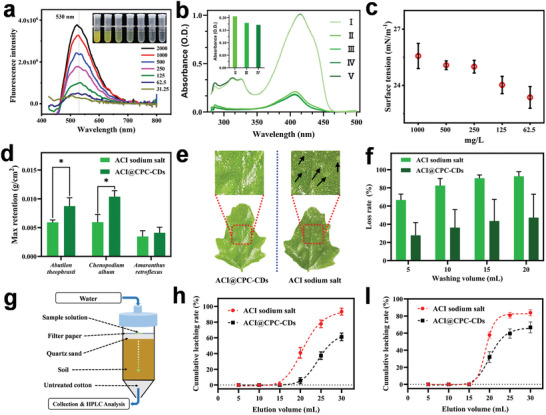
The physical and chemical characteristics of ACI@CPC‐CDs. Emission spectra of ACI@CPC‐CDs with different concentrations at an excitation wavelength of 400 nm (a). b) UV‐vis spectra of DPBF in DMF solution under a UV 365 light source (6 W) at 120 s (I: DPBF only; II: DPBF with protoporphyrin IX; III: DPBF with protoporphyrin IX and CPC‐CDs; IV: DPBF with protoporphyrin IX and ACI@CPC‐CDs; V: DPBF with CPC‐CDs). The insert is the corresponding absorptions at 411 nm of DPBF in DMF solution under different treatments. c) The surface tensions of ACI@CPC‐CDs with different concentrations. d) Maximum retentions of ACI and ACI@CPC‐CDs on different plant leaves. e) The wetting behavior of the aqueous solutions or suspensions of ACI and ACI@CPC‐CDs (the concentration of AIs being 500 mg L^−1^) on the *Chenopodium album* leaves. f) The loss rates of ACI and ACI@CPC‐CDs from *Abutilon theophrasti* leaves after being washed by simulated rain. g) Schematic of leaching experiments in different soils. Cumulative leaching rates of ACI and ACI@CPC‐CDs in Inner Mongolia (h) and Beijing (I) soils.

*Fluorescence characteristic*: Emission spectra of ACI@CPC‐CDs with different concentrations at an excitation wavelength of 400 nm are displayed in **Figure** **6**a. It was obvious that the fluorescence intensity of ACI@CPC‐CDs was dependent on the concentration. With the decrease of the concentrations, the fluorescence intensities of the solution containing ACI@CPC‐CDs at 530 nm were decreased. From another perspective, ACI was also endowed with the fluorescence property after being co‐assembled with CPC‐CDs. Therefore, the light utilization efficiency of ACI could be improved by the light conversion characteristic of CPC‐CDs.

*Detection of singlet oxygen*: Figure 6b and Figure S11 (Supporting Information) illustrate the UV‐vis spectra and absorption intensity of different 1,3‐diphenylisobenzofuran (DPBF) solutions exposed to UV light for 120 s and photographs of *N,N*‐dimethylformamide (DMF) solution with different treatments, respectively. The results showed that the absorption intensities of DPBF solutions (I and V) without Proto IX were significantly higher than those of other treatments (II, III, and IV), indicating that CPC‐CDs couldn't produce singlet oxygen under irradiation. Among the treatments II, III, and IV, DPBF treated with Proto IX and ACI@CPC‐CDs had the lowest absorption (0.171) at 411 nm, followed by DPBF treated with Proto IX and CPC‐CDs (0.179), DPBF treated with Proto IX (0.205). This result might be because the introduction of CPC‐CDs could provide Proto IX with extra light energy to produce more singlet oxygen under ultraviolet light, leading to the degradation of more DPBF. Therefore, the co‐assembly of ACI and CPC‐CDs could promote the activity of ACI with the help of the light conversion of CPC‐CDs.

*Surface activity, maximum retention, and rainfastness*: The surface activity of the spray solution is crucial to the retention and permeation of AIs on target organisms.^[^ ^28^ ^]^ Figure 6c exhibits the surface tensions (mN m^−1^) of ACI@CPC‐CDs with different concentrations. At the concentration range (62.5–1000 mg L^−1^), the surface tensions of ACI@CPC‐CDs suspensions were less than 27 mN m^−1^, which indicated that the formed ACI@CPC‐CDs had excellent surface activity due to the presence of amphiphilic carbon dots. Maximum retentions (*MR*) of ACI sodium salt and ACI@CPC‐CDs on different plant leaves are shown in Figure 6d. The result indicated that the *MR* of ACI@CPC‐CDs was significantly higher than that of ACI sodium salt on the weed leaves, except for *Amaranthus retroflexus*. Particularly on the hydrophobic leaves of *Chenopodium album*, ACI@CPC‐CDs showed an increase of 74.7% in *MR* compared to ACI sodium salt. The desirable wetting function of ACI@CPC‐CDs on the leaf of *Chenopodium album* is also demonstrated visually in Figure 6e. Based on the exceptional surface activity, the rainfastness of ACI@CPC‐CDs on *Abutilon theophrasti* leaves was explored. As shown in Figure 6f, the loss rate of ACI sodium salt was more than 92% after being rinsed with 20 mL of the simulated rainwater. While the loss rate of AIs in the ACI@CPC‐CDs was lower than 48%. This result might be attributed to the excellent activity and high positive charge of ACI@CPC‐CDs, promoting the retention of AIs and enhancing the performance against rainfastness wash‐off.^[^ ^29^ ^]^ Therefore, the ACI@CPC‐CDs with excellent surface activity could improve the retention and adhesion of ACI on the surface of weed leaves, resulting in high utilization of AIs by reducing the loss of off‐target.

*Soil leaching*: According to our previous study, the composition and physicochemical properties of Inner Mongolia (INM) soil and Beijing (BJ) soil are displayed in Table S1 (Supporting Information), which respectively represent loamy sand and loam.^[^ ^15^, ^28^ ^]^ To assess the mobility of ACI@CPC‐CDs in soil, the schematic of the leaching experiment is shown in Figure 6g. The cumulative leaching rates of ACI in INM soil and BJ soil are shown in Figure 6h,I, respectively. The results showed that although ACI sodium salt and ACI@CPC‐CDs had the same leaching trend in two soil samples, the leaching of AIs in ACI@CPC‐CDs clearly presented a delay comparison with free ACI sodium salt. When the leachate reached 30 mL, the cumulative leaching rates of ACI sodium salt in INM and BJ soils were 93.26% and 83.88%, respectively, while those of ACI@CPC‐CDs in the soil samples were 61.16% and 66.72%, respectively. Therefore, the soil mobility of ACI could be retarded by the assembly with CPC‐CDs, which could contribute to the reduction of the potential threats caused by ACI to the aquatic environment.

### 2.6 Biological Activity of ACI@CPC‐CDs

**Figure 7:**
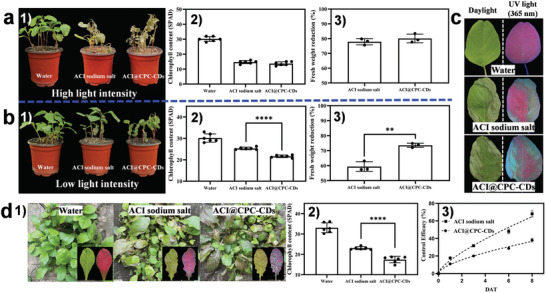
The effects of ACI sodium salt and ACI@CPC‐CDs on *Amaranthus retroflexus* seedlings at three days after treatment under high (a, 5245 LUX) and low light intensity (b, 259.6 LUX) (1): appearance character; 2) chlorophyll content; 3) fresh weight reduction). c) Pictures of the leaves of *Amaranthus retroflexus* seedlings treated by ACI sodium salt and ACI@CPC‐CDs under low light intensity (259.6 LUX) at daylight and UV light (365 nm). The effects of ACI sodium salt and ACI@CPC‐CDs on *Youngia japonica* in the field (1): appearance character at 6 days after treatment (the insert is photographs of the typical leaves in daylight and UV light (365 nm)); 2): chlorophyll content at 6 days after treatment; 3): control efficacy).

*Greenhouse and field experiments*: **Figure** **7**a,b shows the herbicidal activities of ACI sodium salt and ACI@CPC‐CDs against *Amaranthus retroflexus* (*A. retroflexus*) at the concentration of the AIs of 100 mg L^−1^ under high and low light intensities. Under high light intensity, there was no significant difference in the chlorophyll content and fresh weight reduction (*FWR*) of *A. retroflexus* between treatments by ACI sodium salt and ACI@CPC‐CDs. However, under low light intensity, the chlorophyll content of *A. retroflexus* treated with ACI sodium salt was significantly higher than that treated by ACI@CPC‐CDs, which illustrated that the efficacy of ACI was closely related to the light intensity and the efficacy of ACI@CPC‐CDs was affected relatively little by the light intensity. This result was also proved by comparing the *FWR* values between treatments by ACI sodium salt and ACI@CPC‐CDs. In the weak light, the *FWR* value treated by ACI sodium salt was 59.36%, while that treated by ACI@CPC‐CDs was 73.55%. Figure 7c shows the leaves of *A. retroflexus* treated with ACI sodium salt and ACI@CPC‐CDs under low light intensity at daylight and UV light (365 nm). It's easy to find that the damaged area of the leaves of *A. retroflexus* treated by ACI@CPC‐CDs was larger than that treated by ACI sodium salt. Figure 7d shows the herbicidal activities of ACI sodium salt and ACI@CPC‐CDs against weeds in the field at the concentration of AIs being 90 g AI ha^−1^. Combined with the appearance characters and chlorophyll contents of the treated weeds, the control efficacy (68%) of ACI@CPC‐CDs was significantly higher than that (38%) of ACI sodium salt at 8 days after treatment. The results of greenhouse and field experiments indicated that the biological activity of ACI could be enhanced when it was bounded with CPC‐CDs via a co‐assembly strategy.

**Figure 8:**
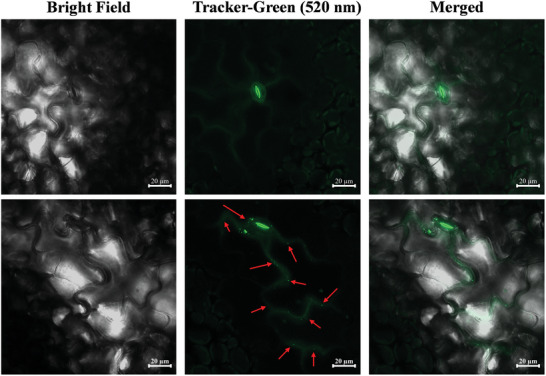
Confocal images of *Amaranthus retroflexus* leaves sprayed with ACI sodium salt (upper) and ACI@CPC‐CDs (under) after 2 h.

*Translocation in the leaf of weed*: Figure S12 (Supporting Information) shows the confocal images of ACI@CPC‐CDs suspensions on the glass slide. Under the excitation of 405 nm, the green fluorescence signal was emitted from ACI@CPC‐CDs, which meant that the fluorescence properties of CPC‐CDs in the ACI@CPC‐CDs were retained and detectable. Moreover, the translocation of ACI@CPC‐CDs in the leaves of *A. retroflexus* is shown in **Figure** **8**. The results showed that only spontaneous fluorescence of stomata and weak fluorescent signals from cell intervals and cell walls could be observed in the leaves with ACI sodium salt. When the leaves were exposed to ACI@CPC‐CDs, clear and strong green fluorescence signals appeared around the stomata, and the point‐shape green fluorescence signals distributed in cell intervals were also found. Therefore, ACI@CPC‐CDs could enter the weed leaves through the stomata and undergo a long‐distance transport in the cell intervals.

**Figure 9:**
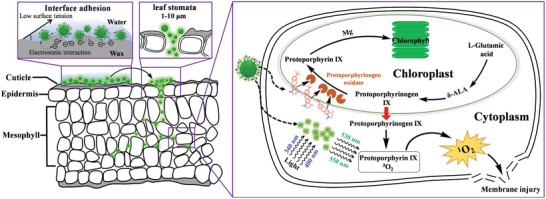
Schematic diagram of the possible mechanism of action of ACI@CPC‐CDs on the weeds.

*Cell membrane permeability*: The effects of ACI sodium salt, CPC‐CDs, and ACI@CPC‐CDs on cell membrane permeability of *A. retroflexus* under low light intensity are presented in Figure S13 (Supporting Information). The relative conductivity (*RC*, uS cm^−1^) as an index of membrane permeability was determined in *A. retroflexus* at twenty‐four hours after treatment. The *RC* value for ACI@CPC‐CDs was the largest (52.90), followed by CPC‐CDs (35.77), ACI sodium salt (34.48), and water (28.34). Obviously, ACI@CPC‐CDs could change the membrane permeability of *A. retroflexus* more than other treatments, causing solute leakage from the plant cells. This result might be ascribed to the generation of more singlet oxygen by the light conversion of CPC‐CDs in the ACI@CPC‐CDs under low light intensity, thus damaging the structure of the leaf cell membrane. Based on the above results, a schematic diagram of the possible action mechanism of ACI@CPC‐CDs on the weeds is presented in **Figure** **9**.

### 2.7 Safety of ACI@CPC‐CDs

**Figure 10:**
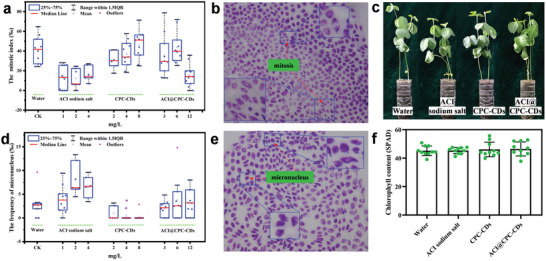
Genotoxicities of ACI sodium salt, CPC‐CDs, and ACI@CPC‐CDs against *Vicia faba*: the mitotic index (a) the frequency of micronucleus d). Photomicrograph (400×) of mitotic (b) and micronucleus (e) in the root tip cells of *Vicia faba* treated by ACI@CPC‐CDs. Pictures (c) and chlorophyll content (f) of soybean seedlings treated with ACI sodium salt (800 mg L^−1^), CPC‐CDs (1440 mg L^−1^), and ACI@CPC‐CDs (2240 mg L^−1^) after four days.

*Genotoxicity evaluation*: The genotoxicities of ACI sodium salt, CPC‐CDs, and ACI@CPC‐CDs at different concentrations against the root tip cells of *Vicia faba* (*V. faba*) are illustrated in **Figure** **10**. According to Figure 10a,d, ACI sodium salt had lower mean values of the chromosome mitotic index (*CMI*) and higher micronucleus frequency (*MNF*) than the blank control group, indicating that ACI sodium salt had certain genotoxicity to the root tip cells of *V. faba* in the experimental concentration range. The mean values of *CMI* and *MNF* treated by CPC‐CDs were at a similar level as those of water, which meant that CPC‐CDs were relatively safe for the plant cell. The mean values of *CMI* of ACI@CPC‐CDs treatment at the concentrations of 2.8 and 5.8 mg L^−1^ also showed the same level as the blank control group. But at a higher concentration (11.2 mg L^−1^), ACI@CPC‐CDs treatment had a negative impact on the *CMI* of *V. faba*. The *MNF* values of *V. faba* treated by ACI@CPC‐CDs at the different concentrations were not significantly increased compared with the water control. Typical patterns of chromosome division and micronucleus in the root tip cells of *V. faba* treated by ACI@CPC‐CDs at a concentration of 11.2 mg L^−1^ are displayed in Figure 10b,e, respectively. Therefore, CPC‐CDs co‐assembled with ACI did not bring a new risk to plant cells and might even alleviate the toxicity of ACI to plants.

*Safety assessment of soybean seedlings*: ACI was widely used in soybean fields against broadleaf weeds. Soybean was selected as a representative plant to evaluate the safety of ACI@CPC‐CDs to non‐target plants. As shown in Figure 10c, no sign of visible damage to the soybean seedlings treated by ACI sodium salt, CPC‐CDs, and ACI@CPC‐CDs was found. Moreover, there was no distinct difference in the chlorophyll content of soybean seedlings among the different treatments (Figure 10f). Therefore, ACI@CPC‐CDs presented good safety to the non‐target plants.

*Effects on soil enzyme activity*: Soil urease is an important component of the soil ecosystem and its activity can reflect the quality and health of the soil environment. The responses of urease activities under the treatments of ACI sodium salt (10 mg kg^−1^ wet soil), CPC‐CDs (20 mg kg^−1^ wet soil), and ACI@CPC‐CDs (30 mg kg^−1^ wet soil) are displayed in Figure S14 (Supporting Information). The results showed there was no obvious difference in urease activity treated with ACI sodium salt, CPC‐CDs, and ACI@CPC‐CDs compared with the control after 14 days of incubation, suggesting that the ACI@CPC‐CDs co‐assembled by ACI and CPC‐CDs were safe to the soil.

## 3 Conclusion

In this work, amphiphilic cationic CDs prepared in a simple and green way were used to fabricate a co‐assembled nano‐system with the typical PPO inhibitor ACI via noncovalent molecular recognition for delivering efficient herbicides. First, CDs with desirable physicochemical properties such as appropriate hydrophobicity, strong fluorescence intensity, high zeta potential, low surface tension, and small particle size were synthesized by using CPC as the carbon source under ultrasound. Then, the co‐assembly based on ACI and CPC‐CDs at the optimal mass ratio of 1:2 was constructed through electrostatic interactions, hydrophobic effect, and π‐π stacking in an aqueous solution. The obtained fluorescent spherical ACI@CPC‐CDs with an average particle size of 174 nm showed the core‐shell structure, low conductivity, strong positive charges, excellent surface activity, high rainfastness, and decreased leaching potential. Moreover, after being absorbed and transported through the weeds, ACI@CPC‐CDs could enhance the herbicidal activity of ACI by transforming the light to produce more singlet oxygen to damage the leaf cell membrane under low light intensity. Safety evaluation revealed that ACI@CPC‐CDs did not increase the genotoxicity of ACI to *V. faba* and caused harm to soybean seedlings as well as had little risks to the soil environment. Taken together, the proposed co‐assembled nano‐system based on ACI and CPC‐CDs possessed unique physicochemical properties, improved herbicidal activity, reduced soil leaching, and favorable safety to non‐target organisms, which provide a new perspective for the efficient utilization of PPO‐inhibiting herbicides.

## Conflict of Interest

The authors declare no conflict of interest.

## Author Contributions

G.T. performed conceptualization, methodology, investigation, visualization, formal analysis, and wrote original draft and reviewed and edited the final manuscript, and acquired the funding; J.W. performed conceptualization and methodology; J.X. performed formal analysis, investigation, and visualization; Y.L. performed formal analysis, investigation, and visualization; Y.H. performed investigation; Z.Z. performed investigation and visualization; X.Z. performed investigation and visualization; G.H. performed investigation; W.Y. performed formal analysis and visualization; Y.C. performed conceptualization, supervision, project administration and acquired funding also wrote the draft and reviewed and edited the final manuscript.

## Supporting information

Supporting Information

## Data Availability

The data that support the findings of this study are available from the corresponding author upon reasonable request.
