# Supplementary material for: Amphiphilic Cationic Carbon Dots for Efficient Delivery of Light‐Dependent Herbicide
Source: Adv Sci (Weinh). 2024 Aug 21;11(39):2406523. doi: 10.1002/advs.202406523 (PMC11496981; doi:10.1002/advs.202406523)
Supplement: Supplementary file 1 — Supporting Information [file ADVS-11-2406523-s001.docx]

***Supporting Information***

**Amphiphilic Cationic Carbon Dots for Efficient Delivery of Light-dependent Herbicide**

Gang Tang, Jialu Wang, Jianhua Xiao, Yulu Liu, Yuqi Huang, Zhiyuan Zhou, Xiaohong Zhang, Gaohua Hu, Weiyao Yan, and Yongsong Cao*

College of Plant Protection, China Agricultural University, Beijing, China

*Corresponding author: NO.2 Yuanmingyuan West Road, China Agricultural University, Beijing, China, 100193

Telephone: 86-10-62734302. Fax: 86-10-62734302.

Email: caoysong@126.com, caoys@cau.edu.cn

**Table of Contents**

[1. **Chemical and materials** 3](#_Toc167780233)

[2. **Synthesis of CPC-CDs** 3](#_Toc167780234)

[3. **Preparation of ACI@CPC-CDs** 3](#_Toc167780235)

[4. **Characterizations** 4](#_Toc167780236)

[5. **Physicochemical property of ACI@CPC-CDs** 5](#_Toc167780237)

[6. **Biological activity of ACI@CPC-CDs** 7](#_Toc167780238)

[7. **Safety of ACI@CPC-CDs** 9](#_Toc167780239)

[8. **Data analysis** 11](#_Toc167780240)

[9. **References** 11](#_Toc167780241)

[10. **Figures and Table** 14](#_Toc167780242)

1. **Chemical and materials**

Cetylpyridinium chloride monohydrate (CPC) (98%), 1,3-diphenylisobenzofuran (DPBF) (97%), and protoporphyrin IX (Proto IX) (95%) were purchased from Tianjin Heowns Biochemical Technology Co., Ltd. Hydrochloric acid (37% solution in water) was provided from Sinopharm Chemical Reagent Co., Ltd. Sodium hydroxide (NaOH) (96%), Rhodamine B (analytical grade), N, N-dimethylformamide (DMF) (99%), ethanol (99.8%), and phosphoric acid (85% in water) bought from Aladdin Industrial Corporation (Shanghai, China). Acetonitrile (HPLC grade) was provided by J.T. Baker, USA. Ultrapure water (18 MΩ cm^−1^) prepared by a Milli-Q water purification system (Millipore, Milford, MA, USA) was applied for all the experiments. The dialysis bag with a molecular weight cut-off of 3000 Da was supplied by Viskase Co., Ltd (USA). Acifluorfen (ACI) (99% purity) was purchased from Qingdao Hansen Biologic Science Co., Ltd. ACI sodium salt was prepared by reacting ACI with NaOH in our laboratory.

1. **Synthesis of CPC-CDs**

The CPC-CDs were synthesized according to the previous methods with some modifications. ^[1,2]^ Typically, 0.134 g of CPC was dissolved in 30 mL of ultrapure water in a 50 mL round-bottom flask, followed by the addition of 0.18 g of sodium hydroxide (NaOH). Then the mixture was quickly transferred to an ultrasonic cleaning machine (Mojie S6000, 40 KHz, 150 W, China) and had ultrasonic treatments with different times (0, 10, 20, 30, 40, 50, 60, 70, 80, 90, 100, 240 min). During the ultrasonic processing, the water temperature in the machine was kept 23℃. After the treatment, the reaction solution was adjusted to pH = 7 with 2 M HCl by A UB-7 acidity meter (Beijing Chenxi Yongchuang Technology Co., Ltd., China). The obtained brown solution was placed in a dialysis bag, which was dialyzed (MW 3000) against ultrapure water for 24 h and then freeze-dried to gain the solid sample. The relative quantum yield (*QY*) of CPC-CDs was measured using rhodamine B (ethanol as the solvent, *QY* = 89%) as a reference according to a method described in the previous work.^[3]^

1. **Preparation of ACI@CPC-CDs**

CPC-CDs obtained at the ultrasonic treatment for 100 min were used to prepare the ACI@CPC-CDs nanocomplex and conduct the subsequent experiments. In brief, 2 mL of ACI sodium salt aqueous solution with different concentrations was slowly added drop by drop to a plastic tube containing 18 mL of 4000 mg/L of CPC-CDs suspension under ultrasonic conditions. After that, the solution was purified and dried to obtain ACI@CPC-CDs nanocomplex by the same method for the preparation of CPC-CDs as mentioned above. For getting the best mass ratio between ACI sodium salt and CPC-CDs, the emission spectra of different reaction suspensions in the 10-fold dilution were analyzed at an excitation wavelength of 400 nm. The assembly rate (*AR*) of ACI@CPC-CDs was calculated as follows: *AR* (%) = (*M_1_* - *M_2_*) / *M_1_* × 100%, where *M_1_* represents the initial mass of ACI sodium salt in the solution, and *M_2_* represents the mass of ACI sodium salt out of the dialysis bag after dialysis.

1. **Characterizations**

A Shimadzu HPLC system integrated with a reversed-phase Kromasil ODS C_18_ column (250 mm × 4.6 mm, 5 μm) was used for the analysis of ACI at room temperature. The mobile phase was water with phosphoric acid (eluent A, pH = 2.8) and acetonitrile (eluent B) (30/70, v/v), ran as the program with a constant flow rate of 1.0 mL min^-1^. The injection volume of each sample was 20 μL, and the detection wavelength for the eluate was 290 nm.

The freshly prepared sample suspensions were diluted to meet the requirements of different measurements. The particle average size, conductivity, polydispersity index (PDI), and zeta potential of CPC-CDs and ACI@CPC-CDs were performed on a laser particle size analyzer (Mastersizer 3000, Malvern Instruments Co. UK). Transmission electron microscopy (TEM) observation was carried out on a microscope (JEM-F200, JEOL, Tokyo, Japan) by directly adding the sample onto a carbon-coated copper grid.

The prepared CPC-CDs and co-assembled ACI@CPC-CDs suspensions were freeze-dried separately to obtain anhydrous samples. Fluorescence characteristics of all the sample were determined by the fluorescence microplate reader (F200, Tean, Switzerland). About 20 mg of anhydrous samples was dissolved in 600 uL of deuterated chloroform to record the ^1^H NMR and ^13^C NMR spectra by A Bruker AVANCE III HD 500 MHz NMR spectrometer (Bruker, Germany). Fourier transformation infrared (FT-IR) spectra were acquired on a Fourier transform infrared spectrometer (Nicolet iS20, Thermo-Fisher, USA) by using the KBr disc method (the mass ratio of anhydrous sample to KBr being 1:50). The UV/vis absorption spectra of ACI sodium salt with 100 mg/L, CPC with 200 mg/L, CPC-CDs with 200 mg/L, and ACI@CPC-CDs 300 mg/L were measured on a UV/vis spectrophotometer (Genesys180, Thermo-Fisher, USA) in the range of 190-500 nm. The thermal behavior of ACI@CPC-CDs was evaluated by using a STA200 simultaneous thermal analyzer (HITACHI, Japan). Powder X-ray diffraction analysis (PXRD) was performed on ACI@CPC-CDs samples (Bruker D8 Advance X-ray diffractometer with Cu Kα radiation, Germany). The ACI@CPC-CDs samples were analyzed using X-ray photoelectron spectrometer (ESCALAB 250Xi, Thermo-Fisher, USA) to clarify the interactive force between ACI and CPC-CDs. The aggregate behavior of ACI@CPC-CDs in water was assessed by the confocal laser scanning microscopy (LSM 800, Zeiss, Japan) at an excitation wavelength of 405 nm to image autofluorescence.

1. **Physicochemical property of ACI@CPC-CDs**

**Thermal stability**: The thermal stabilities of ACI, CPC, CPC-CDs, and ACI@CPC-CDs were measured using thermogravimetric analyzer from 30 to 600°C at a constant rate of 10 °C/min in an inert atmosphere of nitrogen gas.

**Fluorescence characteristic**: The obtained ACI@CPC-CDs nanocomplex was redispersed in ultrapure water to prepare the tested suspensions with the different concentrations (corresponding to 31.25, 62.5, 125, 250, 500, 1000, and 2000 mg/L of ACI). Then the emission spectra of the suspensions were determined at an excitation wavelength of 400 nm.

**Detection of singlet oxygen**: As a singlet oxygen quencher, 1,3-diphenylisobenzofuran (DPBF) with the maximum absorption wavelength at 411 nm was used to investigate the effect of ACI@CPC-CDs on the generation of singlet oxygen caused by Proto IX through its degradation degree.^[4,5]^ In the experiment, five different N,N-dimethylformamide (DMF) solutions, including (I) DMF solution only containing DPBF (100 μM), (II) DMF solution containing DPBF (100 μM) and Proto IX (1 μM), (III) DMF solution containing DPBF (100 μM), Proto IX (1 μM), and CPC-CDs (0.724 mg/L), (IV) DMF solution containing DPBF (100 μM), Proto IX (1 μM), and ACI@CPC-CDs (1.086 mg/L), and (V) DMF solution containing DPBF (100 μM) and CPC-CDs (0.724 mg/L), were prepared under low light environment, respectively. Then these solutions were irradiated under a UV lamp with Emax = 366 nm for 2 min, and the absorption spectra of the solutions in the range of 280-500 nm were recorded by using a UV-vis spectrophotometer.

**Surface activity**: Referring to literature concerned, the critical micelle concentration (*CMC*) of CPC-CDs and the surface tension of ACI@CPC-CDs suspensions with the different concentrations (corresponding to 62.5, 125, 250, 500, and 1000 mg/L of ACI) were assessed by the Wilhelmy plate method using a JK99B automatic tension meter (Powereach, China, Resolution < 0.05 mN/m) under ambient condition. ^[6,7]^ In brief, before the experiment, the instrument was calibrated and checked with ultrapure water as the test sample to identify its surface tension being in a range of 72-73 mN/m. Then, the clean platinum plate was slowly immersed in the aqueous solutions or suspensions of different samples. The surface tension value of the tested solution was recorded when the equilibrium was established between the liquid surface tension and other related forces. The value of *CMC* was found from the intersection of two straight lines of surface tension γ versus logarithm bulk concentration (log C) of CPC-CDs.

**Maximum retention**: The suspension of ACI@CPC-CDs (750 mg/L, corresponding to 250 mg/L of ACI) and ACI sodium salt solution (250 mg/L) containing Tween 80 (5000 mg/L) were prepared for further use. The leaves of uniform size were chosen from three different weeds (*Abutilon theophrasti*, *Chenopodium album,* and *Amaranthus retroflexus*) for the test. Firstly, the mass (*m_0_*, g) of each leaf was weighed and recorded using an electronic analysis balance. Then, the leaf was completely immersed in the aqueous solutions or suspensions of the different samples at room temperature. After 10 seconds, the leaf was taken out and hung in air for two minutes. Subsequently, the mass (*m_1_*, g) of the leaf was weighed again and recorded. A YMJ-D leaf area meter (Hangzhou Daji Electric Instrument Co., Ltd, China) was used to determine the area (*S*) of every tested leaf. The maximum retention (*MR*) on the leaf of every weed was calculated as follows: *MR* = (*m_1_* - *m_0_*)/2 × *S*. Additionally, the wetting behavior on the *Chenopodium album* leaves of ACI sodium aqueous solution and ACI@CPC-CDs suspension was observed and photographed.

**Rainfastness**: the leaves of *Abutilon theophrasti* with uniform size were cleaned gently with ultrapure water to reduce the foreign substance on the surface and dried naturally. Then, 50 uL of ACI@CPC-CDs suspension (750 mg/L, corresponding to 250 mg/L of ACI) or ACI sodium aqueous solution (250 mg/L) containing Tween 80 (5000 mg/L) was evenly smeared on the leaf that was fixed on the slide. After one hour, the slide with the air-dried leaf was put into a beaker at an angle of 30 degrees. Subsequently, the leaf was washed at a speed of 5 mL/min by simulated rainwater. The simulated rainwater in different time periods was collected and the concentrations of ACI in the water were analyzed by HPLC. The loss rate (%) of AIs was calculated with the following equation: loss rate (%) = *M_2_* / *M_1_* × 100%, where *M_1_* represents the total mass of ACI smeared on the leaf and *M_2_* represents the mass of ACI flushed from the leaf via the simulated rainwater (as determined by HPLC), respectively.

**Leaching**: According to the method described in our previous literature, the leaching properties of ACI sodium salt and ACI@CPC-CDs in different soil were investigated. ^[8]^ Soil samples collected from the surface soil (0-20 cm) in Inner Mongolia (INM) and Beijing (BJ) were dried naturally and passed through a 2-mm meshed sieve. Then, the soil columns with a height of 8.0 cm and a diameter of 2.8 cm were made by filling the soil samples into 50 mL plastic tube. Quartz sand and filter paper were sequentially put in the top of soil column. The untreated cotton was placed in the bottom of soil column. The formed soil column was prewetted with ultrapure water for one hour. After that, 100 uL of ACI sodium salt (2000 mg/L) or 100 uL of ACI@CPC-CDs (6000 mg/L, corresponding to 2000 mg/L of ACI) was added to the top of the soil column, which was eluted with ultrapure water. The collected leachate was analyzed to get the amount of ACI by HPLC after filtering through a membrane filter of 0.22 μm. The cumulative leaching rate (%) of ACI was calculated with the following equation: cumulative leaching rate (%) = *M_2_* / *M_1_* × 100%, where *M_1_* represents the initial total mass of ACI added to soil and *M_2_* represents the mass of ACI eluted from the soil column (as determined by HPLC).

1. **Biological activity of ACI@CPC-CDs**

**Greenhouse experiment**: The herbicidal activity of ACI@CPC-CDs in the greenhouse was evaluated at the Greenhouse Base of China Agricultural University (the Haidian District, Beijing) in November 2023. *Amaranthus retroflexus* (*A. retroflexus*) was selected as a representative broadleaf weed to investigate the herbicidal activity of ACI@CPC-CDs. The weed seeds were grown in the flowerpots (length, 7 cm; width, 7 cm; height, 8 cm) filled with vermiculite and nutrition soil (v/v 1:3). The temperatures and photoperiods during day and night in the greenhouse were about 25 ± 2°C/15 ± 2°C and 13/11 h, respectively. Tap water was used to irrigate the weeds at the growth stages. After emergence, the weeds in per pot were thinned to keep basically identical. ACI sodium salt was dissolved directly in ultrapure water with the addition of 0.5% of Tween 80 (v/v) to prepare the spray solutions. A stock suspension of ACI@CPC-CDs was prepared by ultrapure water without involvement of any adjutant. When *A. retroflexus* was in the 4-6 leaf stage, each pot was sprayed with 3 mL of solution using a microaerosol sprayer. The concentrations of AIs were both 100 mg/L for the treatments with ACI sodium salt and ACI@CPC-CDs. The test weeds were divided into group A and group B, which were put in the high (5245 LUX) and low (259.6 LUX) light intensity environments, respectively. Ultrapure water was sprayed as a blank control. Each treatment was repeated three times. Three days after treatment, the chlorophyll content of the third leaf from the top to the bottom and the fresh weight of the above-ground part of weed seedlings were measured and recorded by a chlorophyll meter SPAD-502 Plus (Konica Minolta, Inc., Osaka, Japan) and electronic analytical balance, respectively. The fresh weight reduction (*FWR*) of the above-ground part of the weed seedlings was employed as the assessment index to evaluate the herbicidal activity, which was calculated as follows: (*W*_a_ − *W*_b_) / *W*_a_ × 100%, where *W*_a_ and *W*_b_ represent the fresh weight of blank control groups and the fresh weight of treatment groups, respectively.

**Translocation in the leaf of weed**: The translocation of ACI@CPC-CDs in the weed was investigated by using laser scanning confocal microscopy (LSCM, LSM 800, Zeiss, Japan) to observe its location in leaves. In this test, *A. retroflexus* seedlings with the 4-6 leaf stage, which were also used to apply in the greenhouse experiment, were sprayed with ACI sodium aqueous solution (200 mg/L) or ACI@CPC-CDs suspension (600 mg/L). Two hours after treatment, a small piece of the third leaf from the top to the bottom of *A. retroflexus* was cut and placed on the glass slice dripped with water, which was directly observed by LSCM after being covered with a coverslip at the excitation wavelengths of 405 nm. The aggregate behavior of ACI@CPC-CDs in water was assessed by the same instrument to image autofluorescence.

**Cell membrane permeability**: To explore the possible mechanisms of ACI@CPC-CDs on the improvement of herbicidal activity, the relative conductivities (*RC*) of leaves of *A. retroflexus* seedlings with the 4-6 leaf stage were investigated after leaf-spraying according to the methods from literature.^[9]^ The concentrations of the spray solutions or suspensions for ACI sodium salt, CPC-CDs, and ACI@CPC-CDs were separately 200 mg/L, 400 mg/L, and 600 mg/L. Ultrapure water was sprayed as a blank control. The treated *A. retroflexus* seedlings were placed in an environment with about a light intensity of 1000 LUX. After 24 h, leaf tissue (0.1 g) collected from the third leaf of *A. retroflexus* (from the top) was plunged in ultrapure water for 12 h, and the conductivity (*C_1_*, uS/cm) of the solution was determined by a conductivity meter (DDS 308F, Shanghai Rex Instrument Factory, China). Then, the solution containing leaf tissues was boiled for 40 min, and the conductivity was determined again (*C_2_*, uS/cm). The *RC* (%) of the different treatments was calculated with the following equation: *RC* (%) = *C_1_* / *C_2_* × 100%.

**Field experiment**: The herbicidal activities of ACI sodium salt and ACI@CPC-CDs in the field were assessed in October 2023. A no-till farmland located in Haidian District of Beijing was chosen as an experimental area, which was covered with the broadleaf weed *Youngia japonica*. The temperatures and photoperiods during day and night in the region were about 9 °C/19 °C and 12/12 h, respectively, and the relative humidity was about 40%. The height of weeds at the developmental phase was between 5 and 15 cm. Each plot was designed to be 1 m^2^ (length, 100 cm; width, 100 cm) and was applied about 60 mL of solution by using a microaerosol sprayer. A separation zone (1 m) was set up in every two plots to avoid mutual influence. According to the recommended concentrations of ACI sodium salt in practical application and the growth situation of weeds, ACI sodium salt and ACI@CPC-CDs with the concentrations of AIs being 90 g AI ha^-1^ were applied in every treatment. Ultrapure water was used as a blank control. Each treatment was repeated three times. Based on the standard formed by the Weed Science Society of America, the field control efficacy of ACI sodium salt and ACI@CPC-CDs against weeds was surveyed visually at 1, 3, 6, and 8 days after treatment by using a scale of 0% (no injury on weed) to 100% (complete weed death). The chlorophyll content at 6 days after treatment was measured by a chlorophyll meter mentioned before.

1. **Safety of ACI@CPC-CDs**

**Genotoxicity evaluation**: Referring to the previous literatures, the genotoxicities of ACI sodium salt, CPC-CDs, and ACI@CPC-CDs were assessed by investigating their effects on the root-tip of broad bean (*Vicia faba*) ^[10,11]^. Firstly, broad bean seeds (Fengdou 6) were soaked in water for one day and then placed in a moist environment to obtain the newly emerged roots with about 1-2 cm in length. Afterward, the root tips were immersed into stock solutions or suspensions of the samples with different concentrations at room temperature for 24 h. Then, the treated roots were cultured in fresh water for another 24 h to go through a recovery period. After these treatments, the roots of *Vicia faba* were cut off from the seedlings and fixed by the mixing solution of ethanol and glacial acetic acid (3:1, v/v) for 24 h under ambient condition. Subsequently, these roots were washed with fresh water three times and hydrolyzed in 1 M HCl at 60 °C for 8 min. Finally, approximately 2 mm of the root tips located in the meristematic region was selected to color by 100 μL of carbol fuchsin for 5 min on the slide after being washed with fresh water. The colored roots were squashed and spread by a coverslip, and then were observed by an optical microscope (Olympus Japan Co. Ltd, Japan). Ultrapure water treatment was served as the control. Each treatment had three replications to ensure the accuracy of the test. The chromosome mitotic index (*CMI*) and micronucleus frequency (*MNF*) of the approximately 1000 root tip cells of *Vicia faba* on each slide were calculated respectively, according to the following equations: *CMI* (%) = *N*_1_/*N*_0_ × 100% and *MNF* (%) = *N*_2_/*N*_0_ × 100%, Where *N*_0_ represents the total number of cells observed in the sample, *N*_1_ represents the number of mitotic cells observed in samples, and *N*_2_ represents the number of micronucleus cells observed in samples.

**Phytotoxicity effects on soybean seedlings**: The safeties of ACI sodium salt, CPC-CDs, and ACI@CPC-CDs to nontarget plants were test on soybean seedlings (medium yellow 37) in the greenhouse of CAU. The growth conditions of soybean seedlings were similar to that of *A. retroflexus* described before. When the soybean seedlings were at the 4-6 leaf stage with the height of about 40 cm, each pot of seedlings was sprayed separately with stock solutions or suspensions (2 mL) of ACI sodium salt with 800 mg/L, CPC-CDs with 1600 mg/L, and ACI@CPC-CDs with 2400 mg/L using a microaerosol sprayer. Four days after treatment, the chlorophyll content of the fourth leaf of soybean seedlings (from the top) was measured by the chlorophyll meter. Ultrapure water treatment was served as the control.

**Soil enzyme activities determination**: Referring to the spectrophotometry method described in previous study, the activity of urease was determined to evaluate the ecotoxicology of ACI@CPC-CDs. ^[12,13]^ Before the experiment began, ultrapure water was added to the air-dried Beijing soil to reach 60% of the maximum water-holding capacity (WHC). About 300 μL of aqueous solutions or suspensions of the testing samples was added into 100.00 g soil (wet weight, wt) and then the mixtures were fully stirred to achieve the final experimental concentrations (10 mg of ACI sodium salt /kg wet soil, 20 mg of CPC-CDs/kg wet soil, and 30 mg of ACI@CPC-CDs /kg wet soil). The treated soils were placed into brown bottles at room temperature. The water content of every soil sample was checked by weighing and adjusted to 60 % WHC by adding ultrapure water every other day. Soils were sampled from each bottle on days 14 after treatment for the following enzyme tests. Ultrapure water was used as a blank control. Each treatment was repeated three times in the study. Soil samples (6 g) and methylbenzene (1 mL) were mixed in a flask with 100 ml volume, followed by a 15 min standing at room temperature. Then, 10% urea solution (10 mL) and citrate buffer (pH = 6.7) (20 mL) were added in turn to the flask. After a gentle shaking, the flask was incubated at 37 °C for 24 h and diluted with 38 °C distilled water to a total of 50 mL. After that, filter liquor (1.5 mL), sodium phenate (2 mL), and sodium hypochlorite (5 mL) were added in turn to a flask with a volume of 25 ml, followed by a 20 min incubation for the chromogenic reaction at room temperature. Finally, this system was diluted to 25 mL with ultrapure water, and the absorbance of the mixed solution at 578 nm was measured using a UV/vis spectrophotometer (Genesys180, Thermo-Fisher, USA) in one hour. The urease activity of every soil sample was assessed as micrograms NH_4_^+^-N produced by per gram of dry soil every 1 h.

1. **Statistical analysis**

Statistical analyses were conducted using SPSS statistical analysis software (version 23.0.; SPSS, Chicago, IL, USA). Data fitting analysis and figures were performed using Microsoft office excel 2021, Origin 2021, and GraphPad Prism software. The data were expressed as the mean ± standard error of the mean from at least three independent experiments (n ≥ 3). Details of the n number can be found in the appropriate figure legend. The significance levels are **P*<0.05, ***P*<0.01, ****P*<0.001, and *****P*<0.0001, analyzed by T test. *P* < 0.05 or less was considered significant.

1. **References**
2. Kaur, N.; Mehta, A.; Mishra, A.; Chaudhary, S.; Rawat, M.; Basu, S. Amphiphilic carbon dots derived by cationic surfactant for selective and sensitive detection of metal ions. Mat. Sci. Eng. C 2019, 95, 72-77.
3. Minervini, G.; Panniello, A.; Fanizza, E.; Agostiano, A.; Curri, M. L.; Striccoli, M. Oil-dispersible green-emitting carbon dots: new insights on a facile and efficient synthesis. Materials 2020, 13, 3716.
4. Kozák, O.; Datta, K. K. R.; Greplová, M.; Ranc, V.; Kašlík, J.; Zbořil, R. Surfactant-derived amphiphilic carbon dots with tunable photoluminescence. J. Phys. Chem. C 2013, 117, 24991-24996.
5. Choi, K.-H.; Wang, K.-K.; Oh, S.-L.; Im, J.-E.; Kim, B.-J.; Park, J.-C.; Choi, D.; Kim, H.-K.; Kim, Y.-R. Singlet oxygen generating nanolayer coatings on niti alloy for photodynamic application. Surf. Coat. Technol. 2010, 205, S62-S67.
6. Tang, J.; Ding, G.; Niu, J.; Zhang, W.; Tang, G.; Liang, Y.; Fan, C.; Dong, H.; Yang, J.; Li, J.; Cao, Y. Preparation and characterization of tebuconazole metal-organic framework-based microcapsules with dual-microbicidal activity. Chem. Eng. J. 2019, 359, 225-232.
7. Li, X.; Zhou, Z.; Huang, Y.; Tang, G.; Liu, Y.; Chen, X.; Yan, G., Huang, H.; Zhang, X.; Wang, J.; Cao, Y. A high adhesion co-assembly based on myclobutanil and tannic acid for sustainable plant disease management. Pest Manag. Sci. 2023, 79, 3796-3807.
8. Zhang, X.; Niu, J.; Zhou, Z.; Tang, G.; Yan, G.; Liu, Y.; Wang, J.; Hu, G.; Xiao, J.; Yan, W.; Cao, Y. Stimuli-responsive polymeric micelles based on cellulose derivative containing imine groups with improved bioavailability and reduced aquatic toxicity of pyraclostrobin. Chem. Eng. J. 2023, 474, 145789.
9. Tang, G.; Tian, Y.; Gao, Y.; Zhou, Z.; Chen, X.; Li, Y.; Yu, X.; Wang, H.; Li, X.; Cao, Y. Supramolecular self-assembly of herbicides with reduced risks to the environment. ACS Nano 2022, 16, 4892-4904.
10. Hazrati, H.; Saharkhiz, M. J.; Niakousari, M.; Moein, M. Natural herbicide activity of Satureja hortensis L. essential oil nanoemulsion on the seed germination and morphophysiological features of two important weed species. Ecotox. Environ. Safe 2017, 142, 423-430
11. Tian, Y.; Tang, G.; Gao, Y.; Chen, X.; Zhou, Z.; Li, Y.; Li, X.; Wang, H.; Yu, X.; Luo, L.; Cao, Y. Carrier-free small molecular self-assembly based on berberine and curcumin incorporated in submicron particles for improving antimicrobial activity. ACS Appl. Mater. Inter. 2022, 14, 10055-10067.
12. Huang, Y.; Wang, H.; Tang, G.; Zhou, Z.; Zhang, X.; Liu, Y.; Yan, G.; Wang, J.; Hu, G.; Xiao, J., Yan, W.; Cao, Y. Fabrication of pH-responsive nanoparticles for co-delivery of fungicide and salicylic acid with synergistic antifungal activity. J. Clean Prod. 2024, 451, 142093.
13. Wang, A.; He, M.; Ouyang, W.; Lin, C.; Liu, X. Effects of antimony (III/V) on microbial activities and bacterial community structure in soil. Sci. Total Environ. 2021, 789, 148073.
14. Tang, G.; Tian, Y.; Gao, Y.; Zhou, Z.; Chen, X.; Li, Y.; Yu, X.; Wang, H.; Li, X.; Cao, Y. Supramolecular self-assembly of herbicides with reduced risks to the environment. ACS Nano 2022, 16, 4892-4904.
15. **Figures and Table**


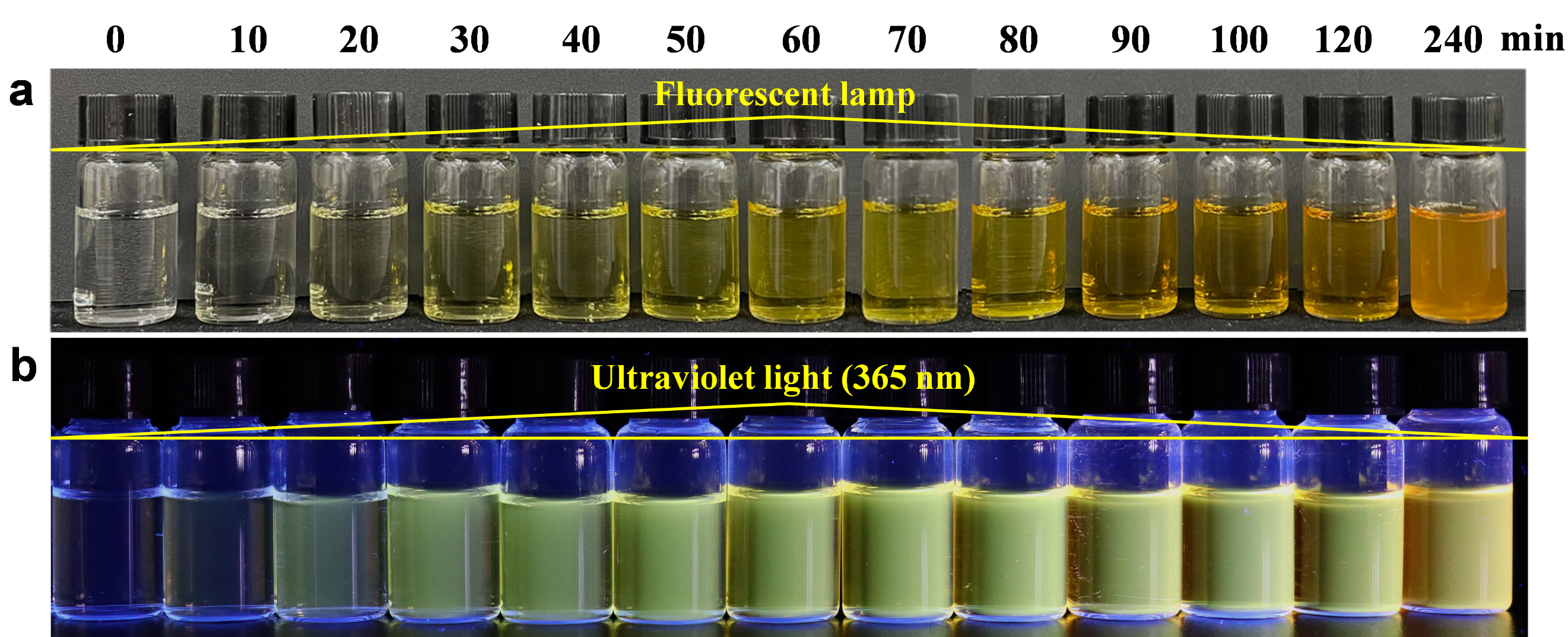


**Figure S1.** Pictures of CPC aqueous solutions containing NaOH after different ultrasonic exposure times under fluorescent lamp (a) and ultraviolet light centered at 365 nm (b).


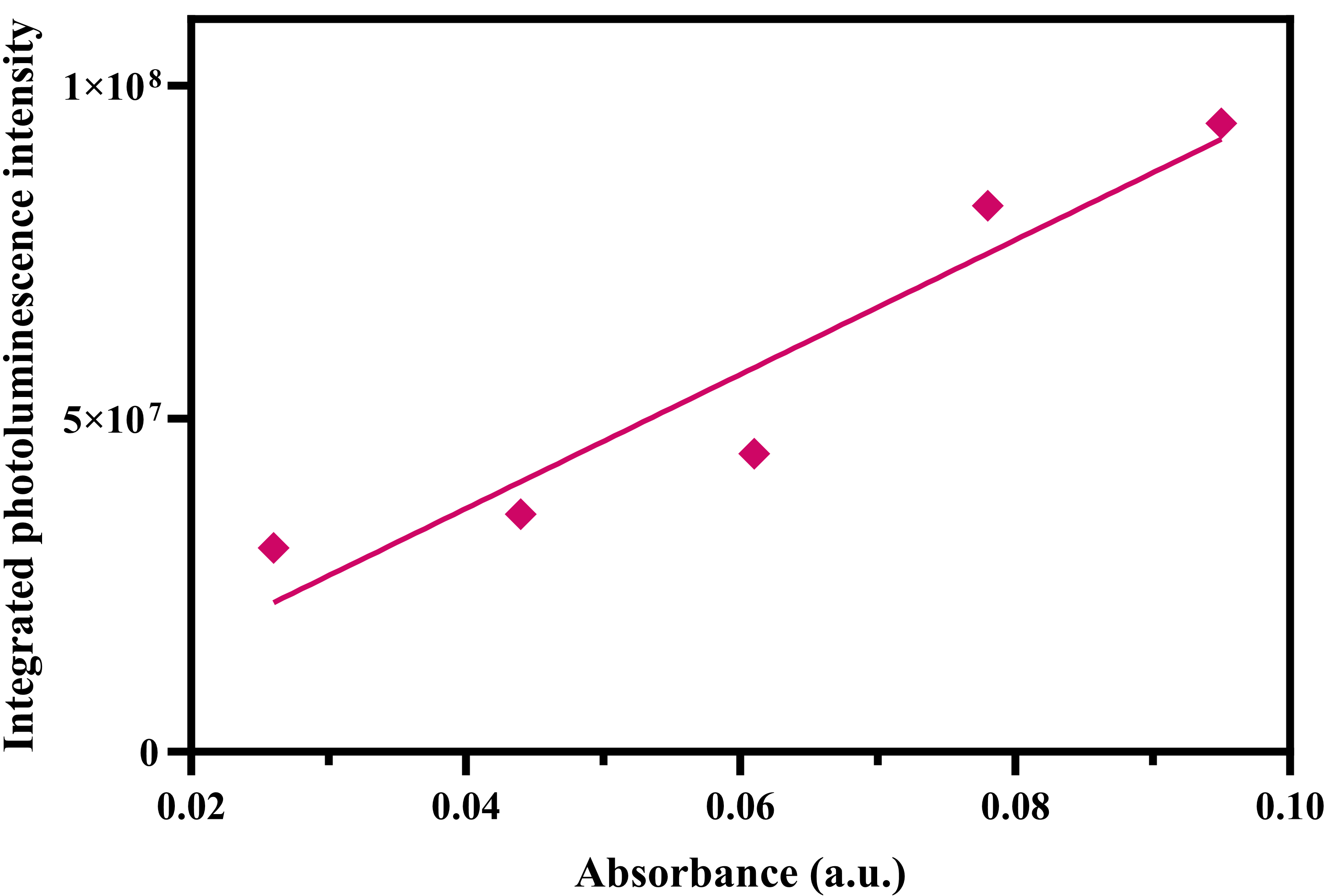


**Figure S2**. Quantum yield of the CPC aqueous solutions containing NaOH after the ultrasonic treatment for 100 min.


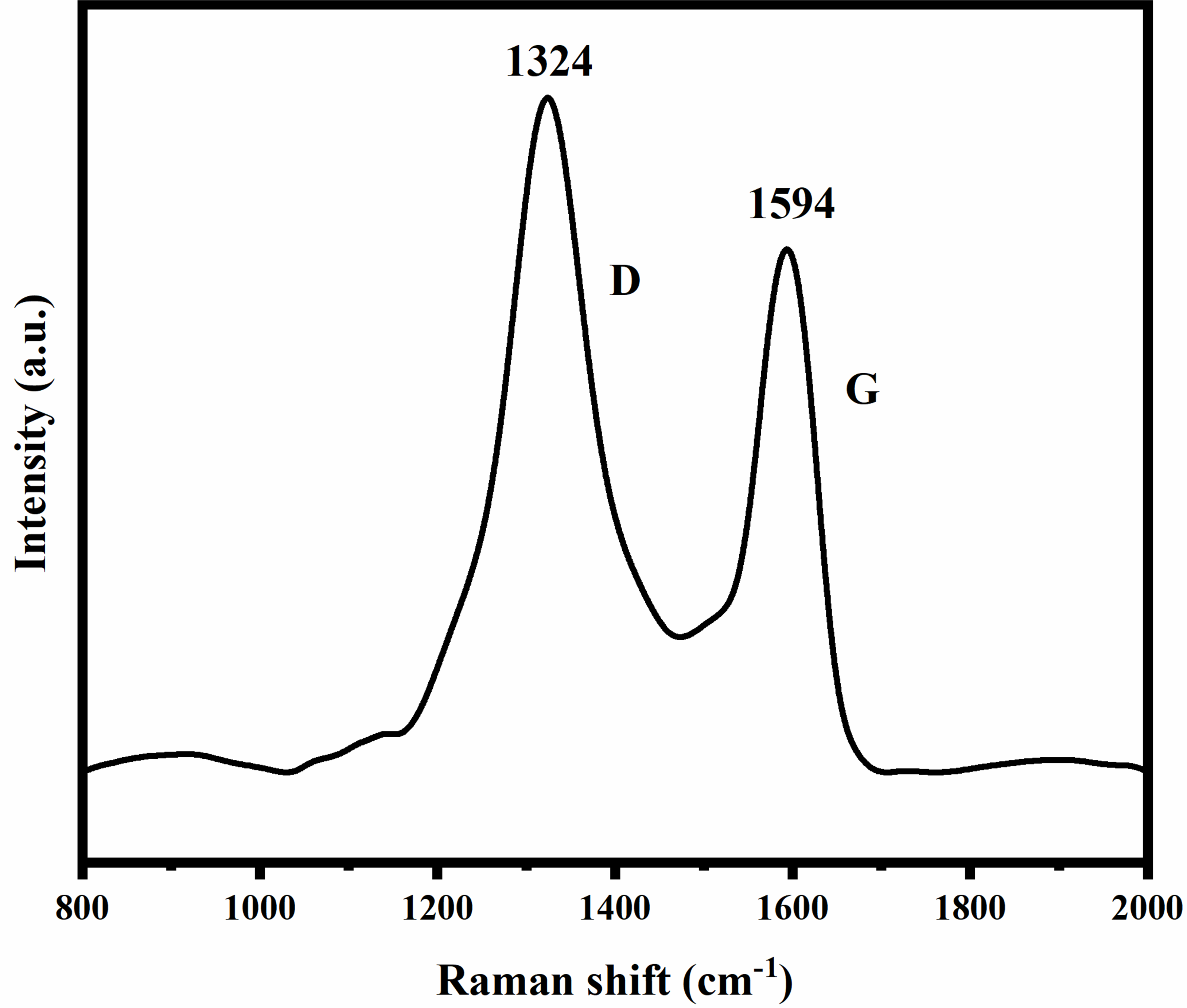


**Figure S3**. Raman spectrum of CPC-CDs obtained after the ultrasonic treatment for 100 min.


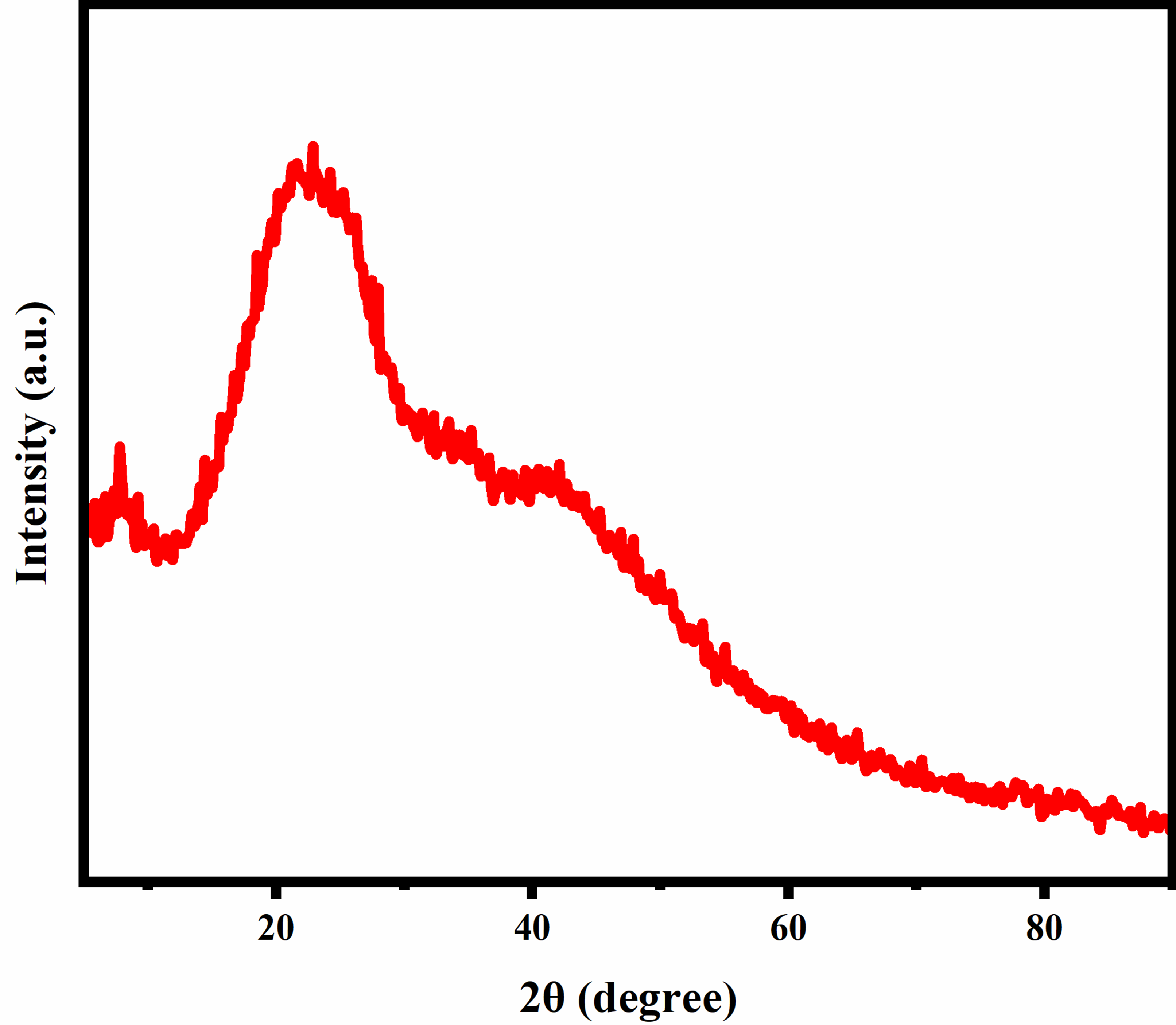


**Figure S4**. X-ray diffraction patterns of CPC-CDs.


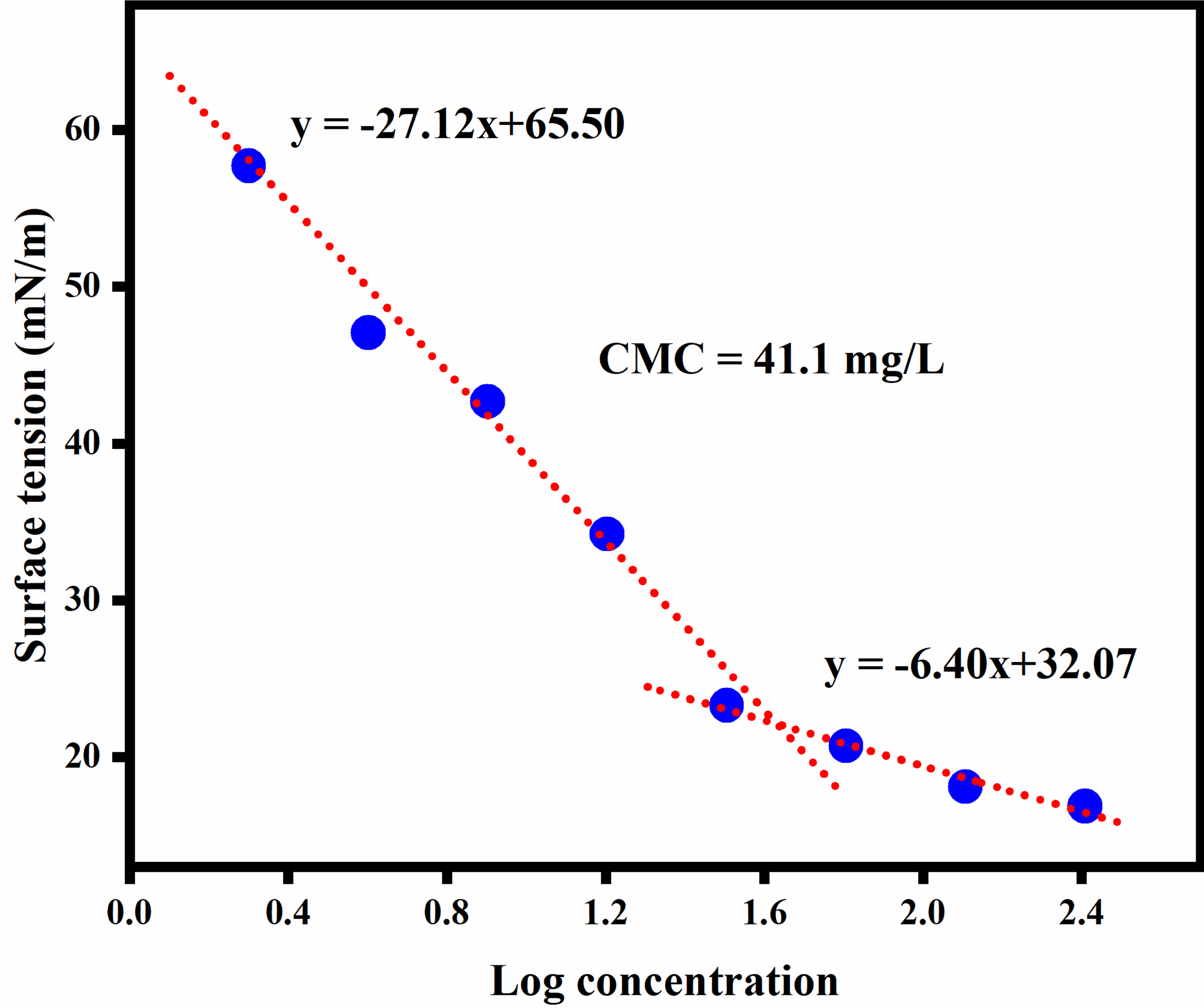


**Figure S5**. Critical micelle concentration of CPC-CDs obtained after the ultrasonic treatment for 100 min.


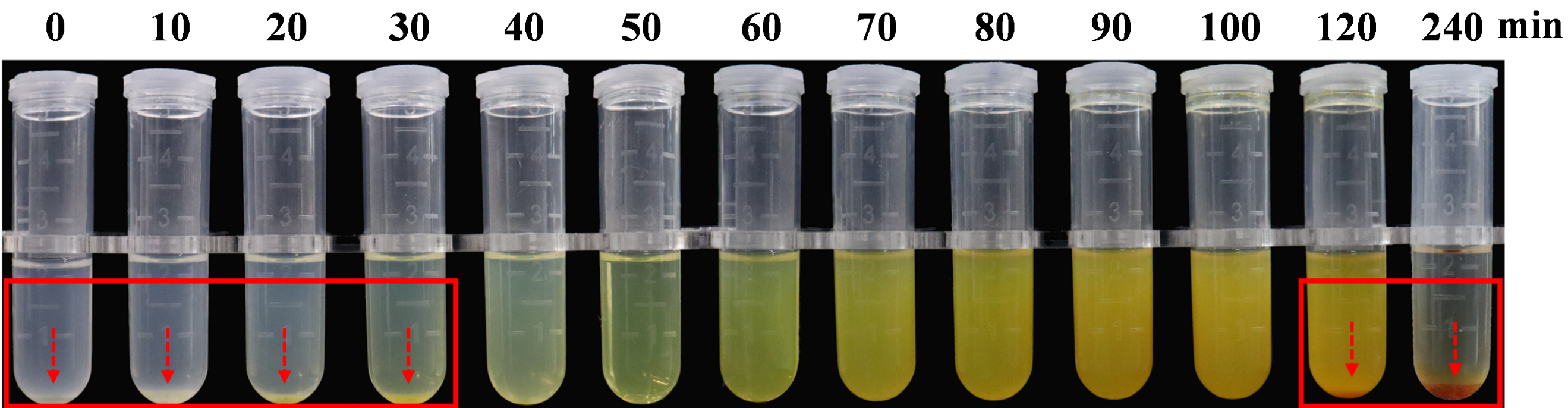


**Figure S6**. Pictures of co-assembly phenomena between CPC aqueous solutions containing NaOH after different ultrasonic exposure times (1.8 mL) and ACI sodium salt solution (0.2 mL, 20000 mg/L).


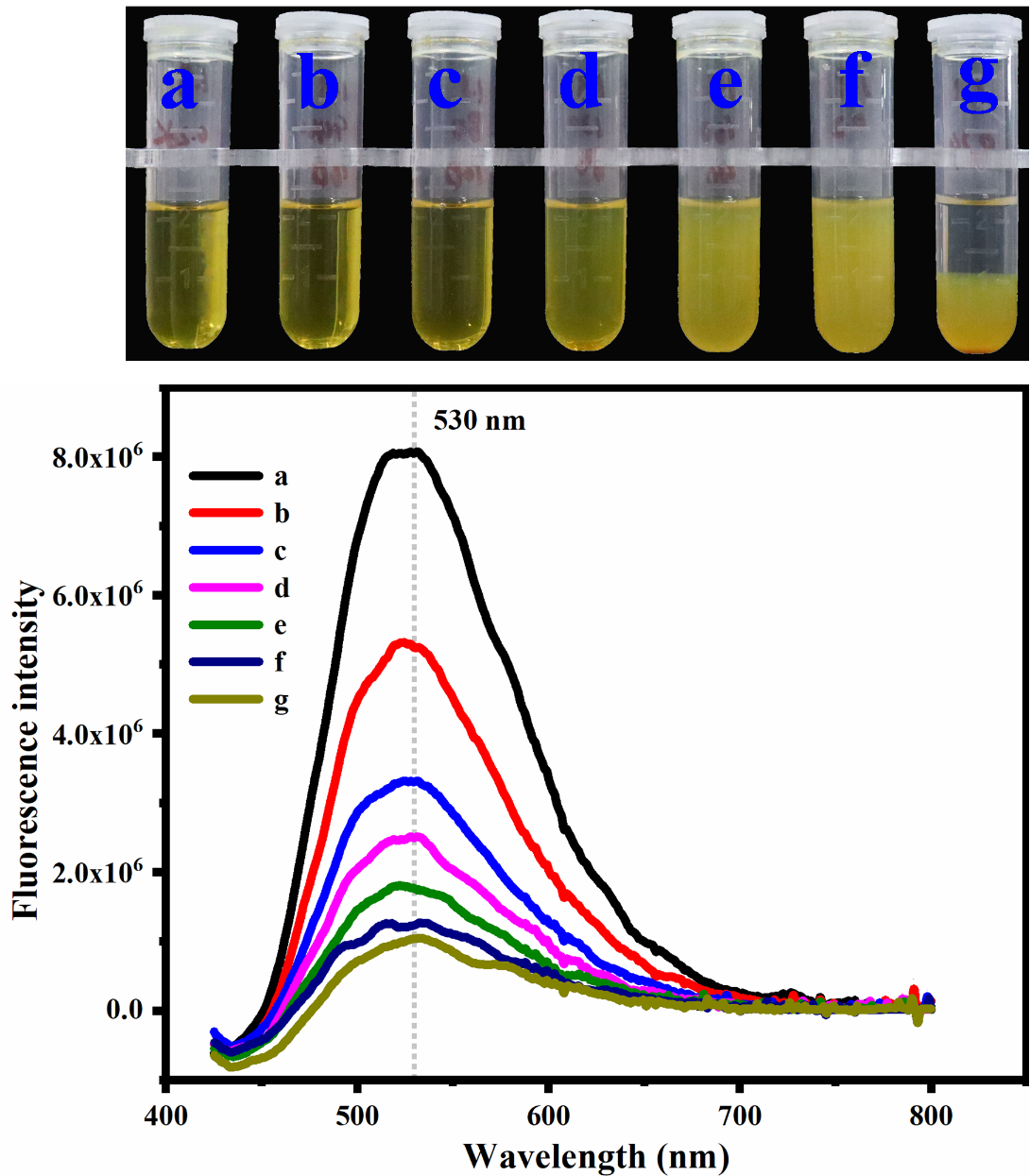


**Figure S7**. Pictures of co-assembly phenomena between 1.8 mL of CPC-CDs suspension obtained after the ultrasonic treatment for 100 min and 0.2 mL of ACI sodium salt solution with different concentrations (a, 0 mg/L; b, 4000 mg/L; c, 8000 mg/L; d, 12000 mg/L; e, 16000 mg/L; f, 20000 mg/L; g, 24000 mg/L) and the corresponding emission spectra at an excitation wavelength of 400 nm.


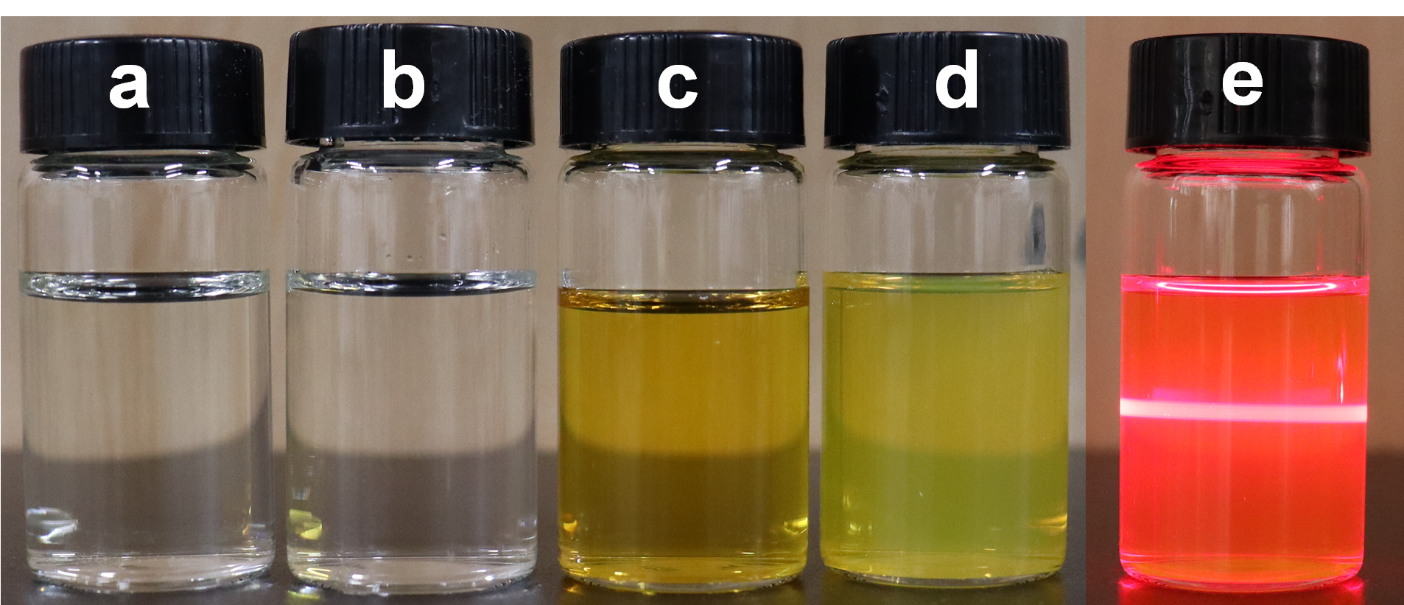


**Figure S8**. Photographs of aqueous solutions or suspensions of ACI sodium salt (a), CPC (b), CPC-CDs (c), and ACI@CPC-CDs NPs (d) as well as the Tyndall effect of the ACI@CPC-CDs NPs in deionized water (e).


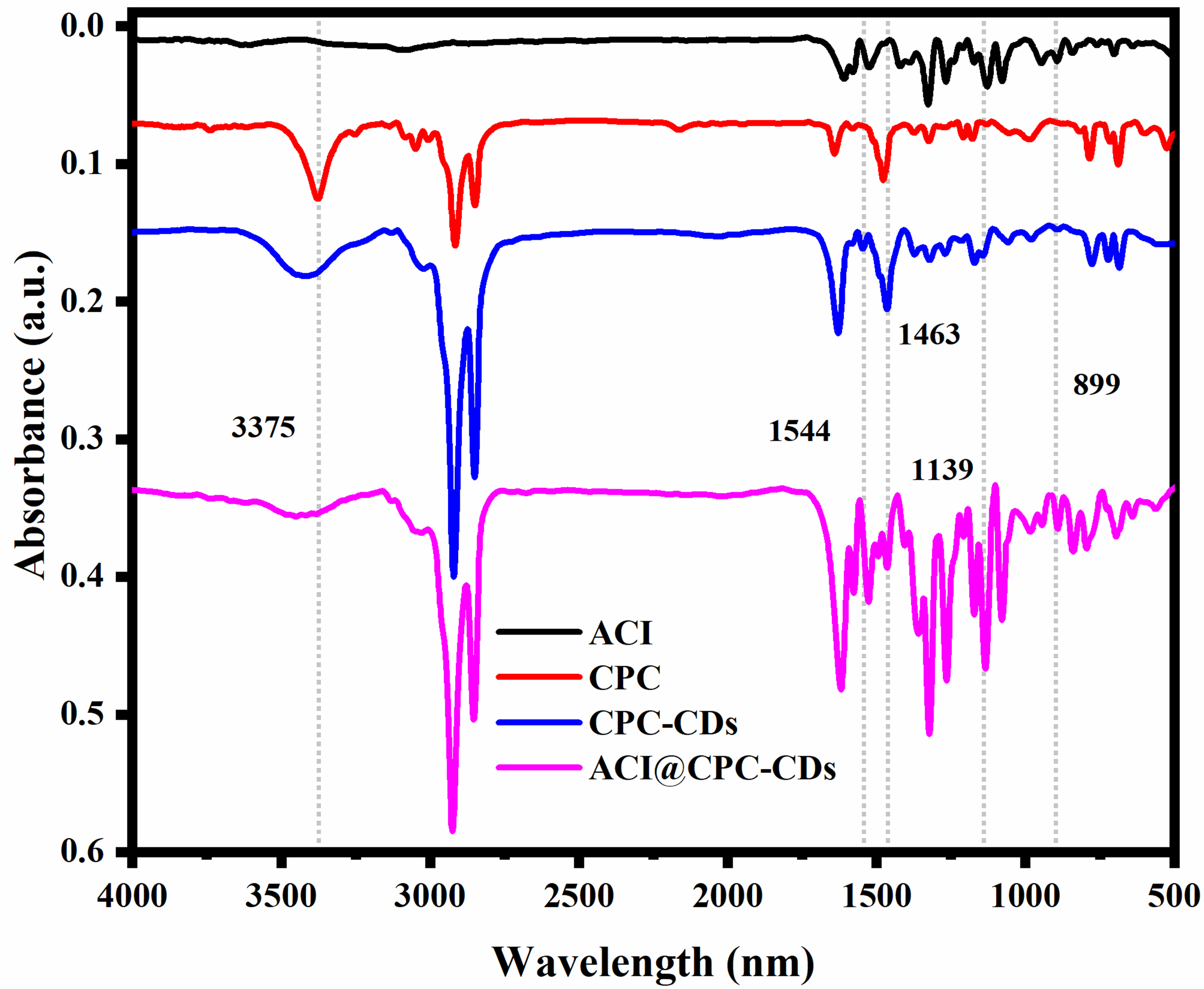


**Figure S9**. FTIR spectra of ACI, CPC, CPC-CDs, and ACI@CPC-CDs.


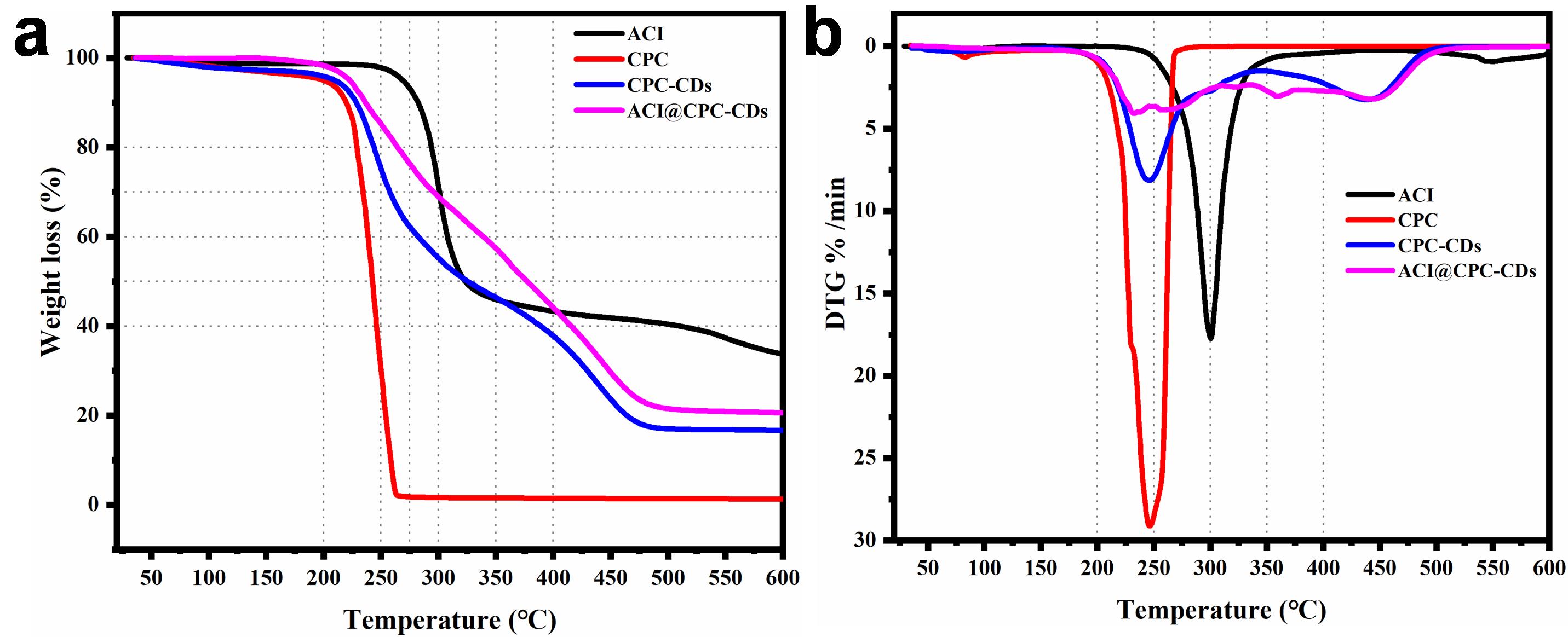


**Figure S10**. TG (a) and DTG (b) cures of ACI, CPC, CPC-CDs, and ACI@CPC-CDs.


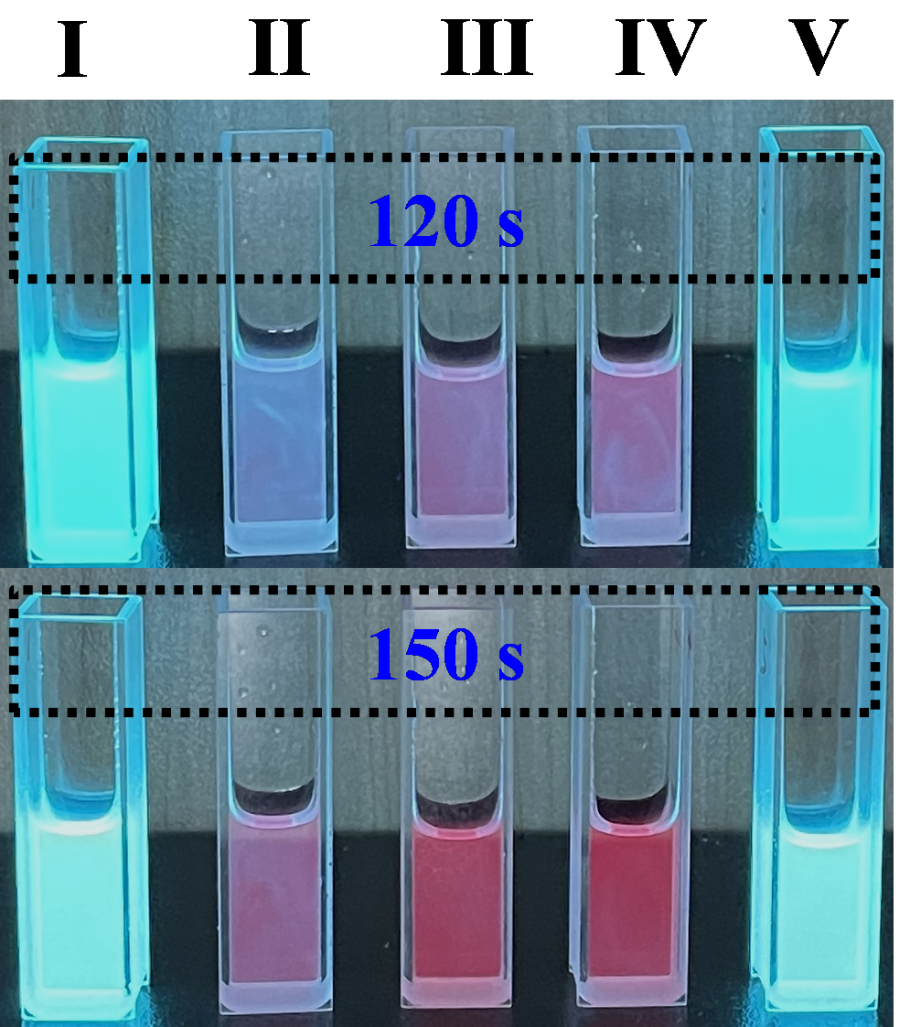


**Figure S11**. Photographs of DMF solutions with different treatments under a UV 365 light source (6 W) at 120 seconds and 150 seconds (Ⅰ: DPBF only; Ⅱ: DPBF with protoporphyrin IX; Ⅲ: DPBF with protoporphyrin IX and CPC-CDs; Ⅳ: DPBF with protoporphyrin IX and ACI@CPC-CDs; Ⅴ: DPBF with CPC-CDs).


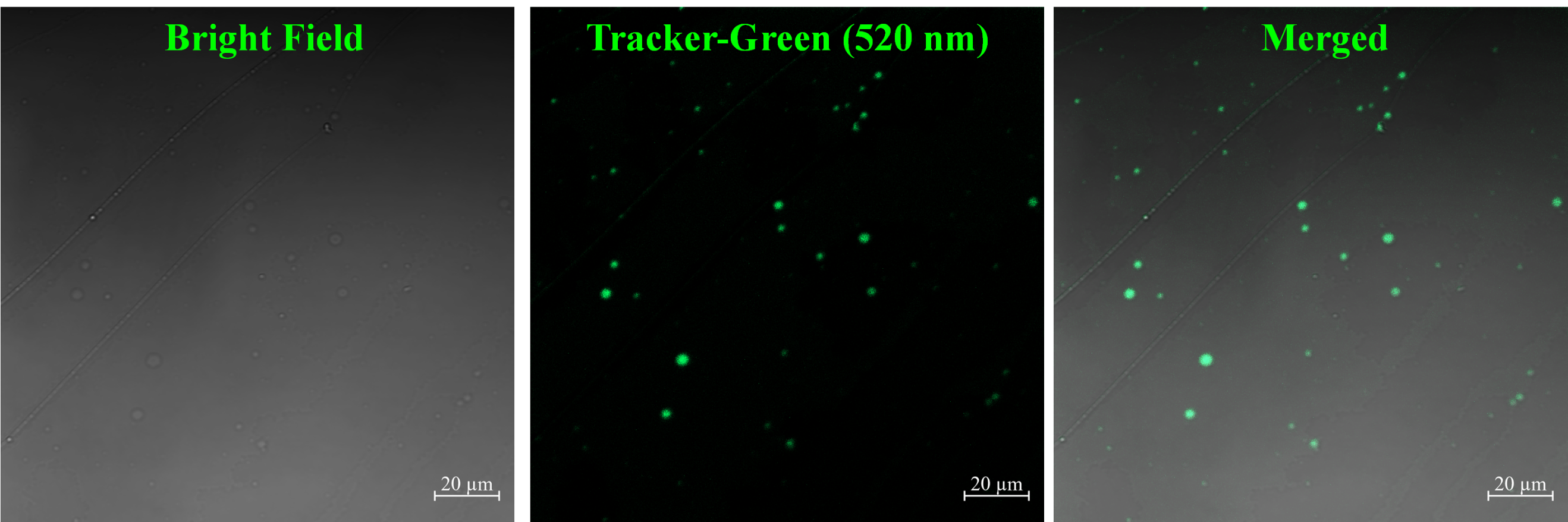


**Figure S12**. Confocal images of ACI@CPC-CDs NPs suspensions on the glass slide.


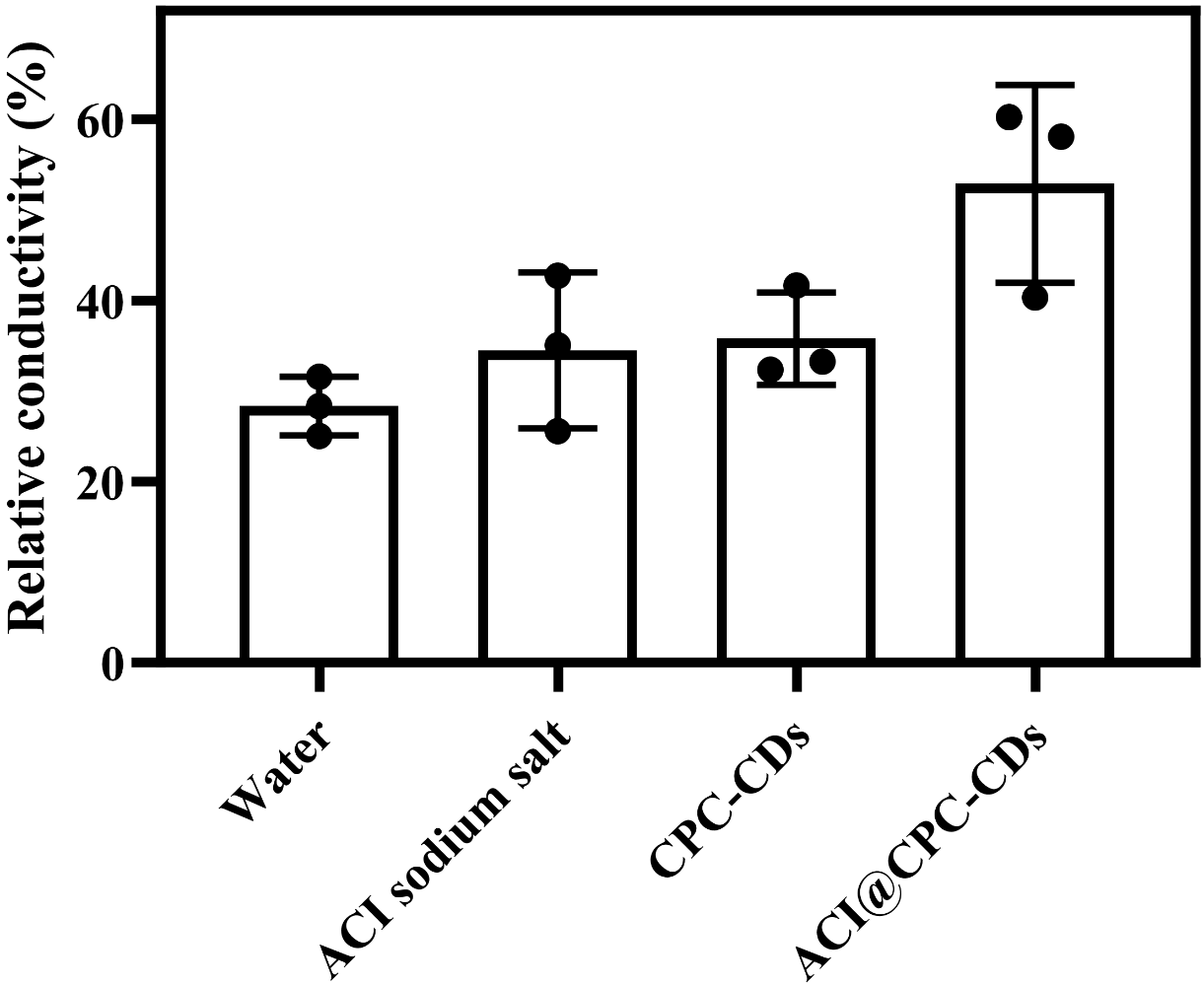


**Figure S13**. The effects of ACI sodium salt, CPC-CDs, and ACI@CPC-CDs on permeability of cell membrane of *Amaranthus retroflexus.*


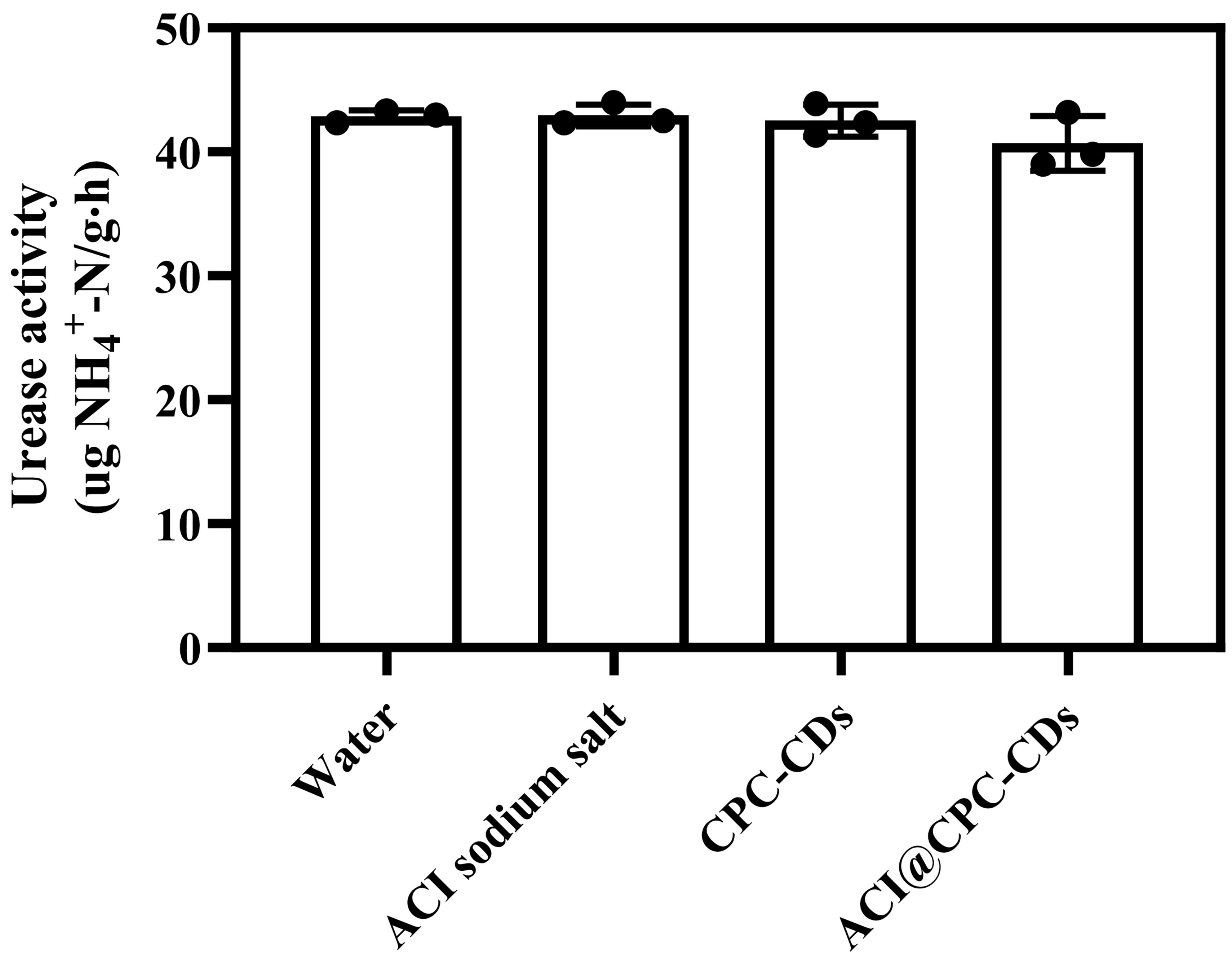


**Figure S14**. Effects of the testing samples on urease activity in soil at 14 days after treatment.

| Site | Soil texture | pH | OC^a^ (%) | CEC^b^ (cmol kg^−1^) | Mechanical composition (%) | | |
| --- | --- | --- | --- | --- | --- | --- | --- |
|  |  |  |  |  | sand | silt | clay |
| Inner Mongolia | loamy sand | 7.46 | 1.01 | 45.2 | 81.32 | 14.58 | 4.10 |
| Beijing | loam | 7.14 | 1.83 | 25.62 | 47.05 | 40.22 | 12.73 |

Table S1. Properties of Inner Mongolia soil and Beijing soil used in leaching experiment.

^a^ Organic carbon content. ^b^ Cation exchange capacity.
